# Transcription factor p73 regulates Th1 differentiation

**DOI:** 10.1038/s41467-020-15172-5

**Published:** 2020-03-19

**Authors:** Min Ren, Majid Kazemian, Ming Zheng, JianPing He, Peng Li, Jangsuk Oh, Wei Liao, Jessica Li, Jonathan Rajaseelan, Brian L. Kelsall, Gary Peltz, Warren J. Leonard

**Affiliations:** 1 Laboratory of Molecular Immunology and the Immunology Center, National Heart, Lung, and Blood Institute, Bethesda, MD 20892-1674 USA; 2 Department of Anesthesia, Stanford University School of Medicine, Stanford, CA 94305 USA; 3 Laboratory of Molecular Immunology, National Institute of Allergy and Infectious Diseases, Bethesda, MD 20892 USA; 4 Present Address: Department of Biochemistry and Computer Science, Purdue University, West Lafayette, IN 37906 USA

**Keywords:** Immunology, Gene regulation in immune cells, T-helper 1 cells, Mucosal immunology

## Abstract

Inter-individual differences in T helper (Th) cell responses affect susceptibility to infectious, allergic and autoimmune diseases. To identify factors contributing to these response differences, here we analyze in vitro differentiated Th1 cells from 16 inbred mouse strains. Haplotype-based computational genetic analysis indicates that the p53 family protein, p73, affects Th1 differentiation. In cells differentiated under Th1 conditions in vitro, p73 negatively regulates IFNγ production. p73 binds within, or upstream of, and modulates the expression of Th1 differentiation-related genes such as *Ifng* and *Il12rb2*. Furthermore, in mouse experimental autoimmune encephalitis, p73-deficient mice have increased IFNγ production and less disease severity, whereas in an adoptive transfer model of inflammatory bowel disease, transfer of p73-deficient naïve CD4^+^ T cells increases Th1 responses and augments disease severity. Our results thus identify p73 as a negative regulator of the Th1 immune response, suggesting that p73 dysregulation may contribute to susceptibility to autoimmune disease.

## Abstract (web summary)

Heterogeneous helper T (Th) cell responses contribute to differential susceptibility to immunological disorders. Here the authors perform haplotype-based computational screens of 16 inbred mouse strains to identify a transcription factor, p73, as an important negative regulator of Th1 differentiation, with p73 deficient mice manifesting alterations in two inflammatory disease models.

## Introduction

T cells play a central role in regulating immune responses. Following antigen stimulation, naive CD4^+^ T cells differentiate into distinct T helper (Th) subsets (e.g., Th1, Th2, Th9, and Th17 cells), with characteristic patterns of cytokine expression and specific functions. Unbalanced Th cell responses result in susceptibility to infectious, allergic, and autoimmune diseases, as well as cancer^1–3^. Moreover, after exposure to an infectious agent or stimulus, individual differences in Th cell responses can influence disease susceptibility and outcome. For example, after *Leishmania* infection, some individuals have a silent infection, whereas others develop non-healing chronic lesions depending on the relative potency of the Th1 versus Th2 response^4^. The quantitative and/or qualitative variations in these responses in different individuals indicate that Th cell differentiation is under complex genetic control. Although the basic molecular mechanisms that initiate Th cell differentiation have been defined, genetic factors that modulate Th cell responses are incompletely understood. To further identify the factor(s) involved, we used haplotype-based computational genetic mapping (HBCGM)^5,6^ to analyze Th1 differentiation data from multiple mouse strains. From this analysis, we identified a p53 family member, Transformation related protein 73 gene (*Trp73*)^7^, as a regulator of Th1 differentiation.

p73 regulates many important cellular functions, including cell cycle progression, apoptosis, genome stability, and metabolism^8–11^. Abnormal p73 expression is often associated with the development of solid tumors and hematological malignancies, including lymphoma^12–15^. Although the role of p73 in cancer has been extensively studied, its role in immune modulation is less well studied. Interestingly, *Trp73*^−/−^ mice develop chronic inflammation, including rhinitis, otitis, periorbital edema, and conjunctivitis^16^, consistent with dysregulated immune responses.

Here we identify p73 as a negative regulator of Th1 differentiation and interferon-γ (IFNγ) production. Moreover, in experimental allergic encephalitis, a mouse model of multiple sclerosis (MS), we find augmented IFNγ expression by cells from *Trp73*^−/−^ mice and show that these animals have decreased disease severity, whereas in a model of adoptive transfer inflammatory bowel disease, *Rag2*^−/−^ hosts receiving of p73-deficient naive CD4^+^ T cells as opposed to wild-type (WT) T cells have augmented Th1 responses as well as greater disease.

## Results

### Identification of p73 as a Th1-related gene

To identify novel genetic factor(s) that modulate T helper cell differentiation, we differentiated naive splenic CD4^+^ T cells from 16 inbred mice strains^17^ into Th1 cells and assessed the extent of Th1 differentiation by measuring mRNA encoding the Th1 signature cytokine, IFNγ, at six time points. There was a substantial difference in the basal mRNA level of *Ifng* and the extent of Th1 differentiation among these 16 strains. For example, SM/J, 129S, and NZB splenocytes had relatively high *Ifng* mRNA expression compared to the other strains at most time points (Supplementary Fig. 1), with strong and significant inter-strain differences in *Ifng* mRNA expression (*P* values were <1E−50, 1E−7, 9E−12, 2E−15, 1E−11, and 3E−6 at 0, 4, 16, 24, 48, and 72 h of Th1 differentiation, respectively), indicating that the observed variations resulted from genetic differences.

To identify the genetic basis for these differences, HBCGM^17,18^ was used to compare the measured *Ifng* mRNA levels with the pattern of genetic variation across the 16 strains analyzed. This analysis identified a broad range of genes (Supplementary Data 1), including 32 transcription factors, 12 transcription cofactors, and 5 chromatin remodeling proteins with a correlated allelic pattern that could potentially affect *Ifng* mRNA expression (*P* < 0.001; Table 1 and Supplementary Table 1), and these 49 genes included several that are known Th1-associated transcription factors (*Stat1*, *Stat4*, and *Irf4)* (Table 1). Of the 49 genes, only 3 (*Rora*, *Stat1*, and *Trp73)* had allelic patterns that were significantly associated with *Ifng* expression at 3 different time points during Th1 differentiation (Supplementary Table 1). *Stat1* is well known to contribute to Th1 differentiation, and *Rora* was previously reported to contribute to Th17 differentiation^19^. *Trp73* was not previously known to play a role in Th cell differentiation, but based on its important roles in the development and progression of cancer, we investigated whether this transcription factor also plays a role in Th1 differentiation. The *Trp73* allelic pattern (i.e., haplotype blocks) significantly correlated with *Ifng* expression at 0, 4, and 24 h during Th1 differentiation (Supplementary Table 1).

**Table 1:** Transcription factors, cofactors, and chromatin remodeling proteins with allelic patterns that are correlated with *Ifng* expression in Th1.

Gene category	Genes (*P* < 0.001)
Transcription factors (*n* = 32)	*Dlx2*, *Ebf1*, *Esr1*, *Etv2*, *Foxd3*, *Foxj2*, *Foxm1*, *Foxo3*, *Glis3*, *Hif1a*, *Irf4*, *Junb*, *Mef2c*, *Myt1l*, *Npas2*, *Npas3*, *Nr3c2*, *Nr6a1*, *Olig3*, *Pbx1*, *Rora*^a^, *Runx2*, *Runx3*, *Satb2*, *Stat1*^a^, *Stat4*, *Tcf12*, *Tcf4*, *Trp73*^a^, *Uncx*, *Vdr*, *Zbtb17*
Transcription cofactors (*n* = 12)	*Csrp1*, *Dcc*, *Greb1*, *Ifi203*, *Lmcd1*, *Mybbp1a*, *Pdlim1*, *Rbbp9*, *Rbpms*, *Sap130*, *Spen*, *Yaf2*
Chromatin remodeling proteins (*n* = 5)	*Cbx7*, *Hdac1*, *Hdac9*, *Itgb3bp*, *Jmjd1c*

Genes were identified by haplotype-based computational genetic mapping, with the pattern of genetic variation significantly correlated with *Ifng* mRNA in Th1 (*P* < 0.001). Genes from category transcription factors, transcription cofactors and chromatin remodeling proteins are shown in this table. *P* values were calculated using analysis of variance (ANOVA)-based statistical modeling. Exact *P* values for each gene are listed in Supplementary Table 1.

^a^Genes with allelic patterns that were correlated with *Ifng* expression at three time points.

### p73 negatively affects Th1 differentiation

**Fig. 1:**
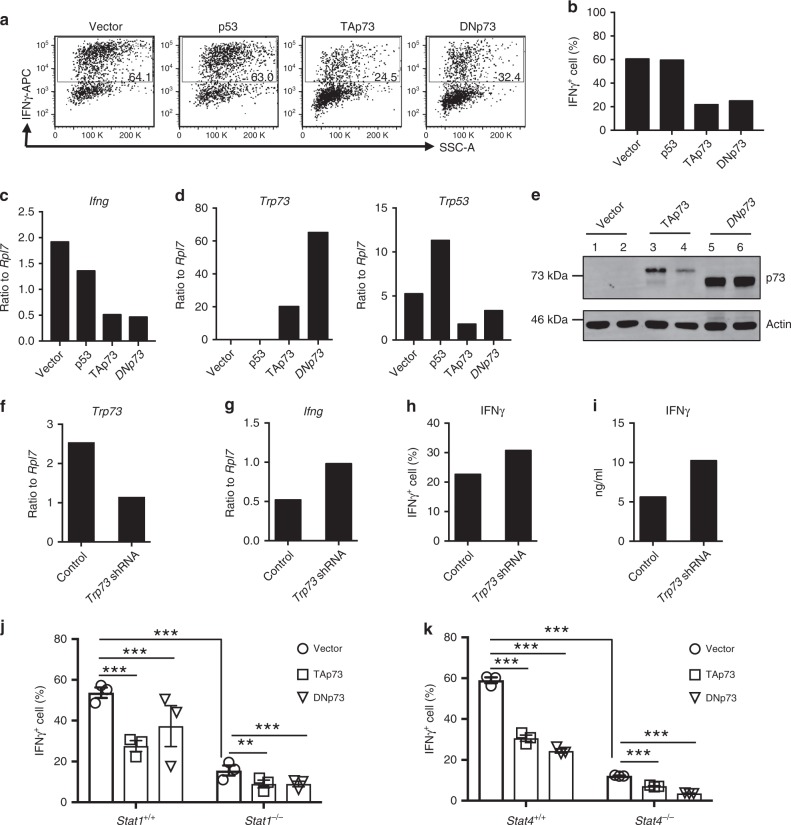
p73 expression level negatively correlated with IFNγ expression during Th1 differentiation. **a**–**e** Empty vector, p53, TAp73, and DNp73 were overexpressed in Th1 cells via retroviral transduction. Transduced cells were identified by GFP^+^ and enriched by FACS sorting. IFNγ protein was assayed by flow cytometric staining (**a**, **b**) and mRNA expression of *Ifng* (**c**), *Trp73* (**d**, left panel), and *Trp53* (**d**, right panel) were assessed by qRT-PCR. All data shown in **a**–**d** are mean values of two biological replicates. **e** p73 protein and control actin levels were detected by western blotting. Lanes 1–2, 3–4, and 5–6 are duplicates for empty vector, TAp73, and DNp73, respectively. A full scan blot image is provided in Supplementary Fig. 9a. **f**–**i** Th1 cells were transduced with constructs containing different shRNAs against *Trp73* or non-targeting control, and the effects of shRNA knockdown on *Trp73* (**f**) and *Ifng* (**g**) mRNA levels were assessed by qRT-PCR. Intracellular IFNγ levels were measured by FACS staining (**h**) and secreted IFNγ levels were assessed by ELISA (**i**). **j** The empty vector, TAp73, and DNp73 were overexpressed in Th1 cells derived from WT or *Stat1*^−/−^ littermate mice (*n* = 3 animals per group). IFNγ expression levels were assayed by intracellular staining followed by flow cytometric analysis. The effects of p73 overexpression on IFNγ protein expression levels are shown as a bar graph of mean values ± SEM. **k** Similar experiments to those in **j** were performed in Th1 cells derived from WT or *Stat4*^−/−^ littermate mice (*n* = 3 animals per group). Experiments **a**–**d** were repeated *n* ≥ 20 times and all other experiments (**e**–**k**) were repeated *n* = 3 times, and representative results are shown. *P* values between specified groups were determined by a two-tailed unpaired Student’s *t* test. **P < 0.01; ***P < 0.001. Source data for **a**–**k** are provided in Source Data Files.

p73 has a complex role in tumorigenesis, with two functionally distinct classes of isoforms in humans. TAp73 isoforms that contain the N-terminal transactivation (TA) domain function as tumor suppressors, whereas N-terminally truncated isoforms (DNp73) lack the TA domain and act as oncogenes that inhibit TAp73 and p53 in a dominant-negative fashion. The balance between TAp73 and DNp73 determines the overall effect of p73 on tumorigenesis^14,20^. To assess the potential effect of p73 on Th1 differentiation, we used retroviral transduction to overexpress TAp73, DNp73, or p53 in mouse Th1 cells. Whereas p53 had no effect, cells transduced with either p73 isoform had significantly reduced IFNγ protein as assessed by intracellular staining (Fig. 1a), a lower percentage of IFNγ^+^ cells (Fig. 1b), and diminished *Ifng* mRNA expression (Fig. 1c). These results indicated that the p73 effect on IFNγ was independent of the presence or absence of its TA domain. We confirmed expression of both p73 and p53 by quantitative reverse transcription polymerase chain reaction (qRT-PCR; Fig. 1d) and of p73 protein by western blotting (Fig. 1e). DNp73 tended to be more highly expressed than TAp73 (Fig. 1e), but relatively similar levels of inhibition of *Ifng* mRNA and IFNγ protein expression were observed with both isoforms (Fig. 1a–c); thus TAp73 might be more potent, but it is also possible that even the lower level of the expressed protein was simply above the threshold required for efficient inhibition. The decrease in IFNγ was not due to either increased apoptosis (Supplementary Fig. 2a, b) or impaired proliferation (Supplementary Fig. 2c) of the transduced Th1 cells.

To further investigate the role of p73 in Th1 differentiation, endogenous p73 gene expression was determined by RNA sequencing (RNA-Seq). Although its expression level was relatively low, p73 was induced after 3 days of Th1 differentiation as compared to naive CD4^+^ T cells (Supplementary Fig. 2d). We next reduced p73 gene expression by retroviral transduction of short hairpin RNA (shRNA) into Th1 cells generated from C57BL/6 splenic T cells (Fig. 1f). This significantly increased *Ifng* mRNA levels (Fig. 1g), the percentage of IFNγ^+^ cells (Fig. 1h), and amount of secreted IFNγ protein (Fig. 1i) in Th1 cells. Collectively, these results indicate that p73 is a negative regulator of Th1 differentiation.

Signal transducer and activator of transcription factor 1 (STAT1) and STAT4 are key transcription factors that are required for normal Th1 differentiation^1,21^ and were both identified as potential factors that affect *Ifng* mRNA expression in our computational genetics analysis (Table 1). STAT1 was reported to physically associate with TAp73, and *Stat1* gene expression is downregulated in *p73*^−/−^ mouse embryonic fibroblasts^22^. Therefore, we investigated whether p73 inhibition of Th1 differentiation is independent of STAT1 or STAT4. As expected, the extent of Th1 differentiation of the *Stat1*^−/−^ or *Stat4*^−/−^ cells was significantly lower than in cells from WT littermates (Fig. 1j, k); however, interestingly, overexpression of either p73 isoform in *Stat1*^−/−^ or *Stat4*^−/−^ T cells further reduced IFNγ^+^ T cells (Fig. 1j, k), indicating that neither STAT1 nor STAT4 by itself was essential for inhibition by p73.

### p73 DNA-binding domain (DBD) is required for Th1 inhibition

**Fig. 2:**
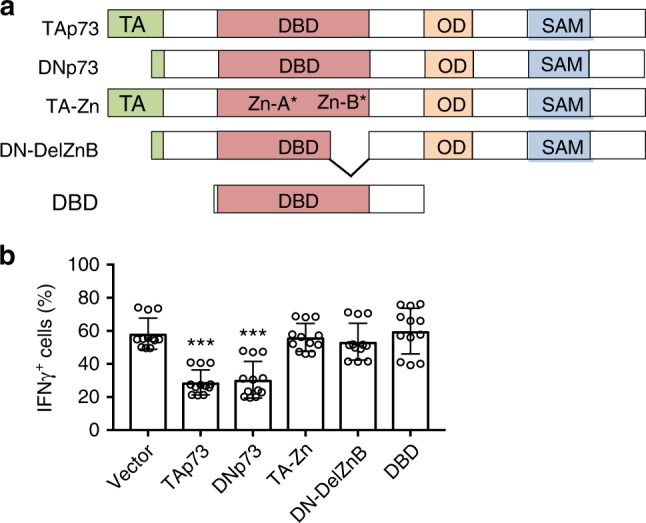
DNA-binding activity of p73 is required for its inhibitory effect on Th1 differentiation. **a** Schematic of the domain structure of WT and mutant p73 proteins. The transactivation (TA) domain, DNA-binding domain (DBD), oligomerization domain (OD), and sterile α motif (SAM) are indicated. Mutations in two zinc-binding sites (Zn-A* and Zn-B*) are marked as indicated. **b** Empty vector or different p73 constructs were overexpressed in Th1 cells via retroviral transduction. IFNγ expression levels were determined by intracellular staining and flow cytometric analysis and expressed as the percentage of IFNγ^+^ cells from different p73 mutant-transduced cells (GFP^+^). Data represent mean ± S.D. from a total of *n* = 12 biological replicates over four independent experiments. *P* values of mutant p73 compared to empty vector were determined by two-tailed unpaired *t* test. ***P < 0.001. Source data for **b** are provided in a Source Data File.

Because both TAp73 and DNp73 inhibited IFNγ expression (Fig. 1a, b), we inferred that the inhibitory effect of p73 on IFNγ production does not require its TA domain. Rather, it depends upon intrinsic properties that are shared by both isoforms, and we therefore investigated the role of DBD. The p73 DBD contains two zinc-binding sites, one of which is in α-helix H1 (Zn-A) and the other is in loop L3 (Zn-B)^23^, and zinc binding is crucial for the DNA-binding ability of p73^24^. We constructed p73 DBD mutants with either point mutations in both zinc-binding sites in TAp73 (TA-Zn) or internal deletion of the Zn-B region in DNp73 (DN-DelZnB), as well as a mutant containing only the DBD (Fig. 2a). When we transduced WT TAp73, DNp73, and each of these mutant constructs into Th1 cells and gated on the transduced cells, only the TAp73 and DNp73 parental constructs could inhibit IFNγ expression or Th1 differentiation (Fig. 2b), indicating that the DNA-binding activity of p73 is required for its inhibition of Th1 differentiation.

### Identifying p73 target genes in Th1 cells

**Fig. 3:**
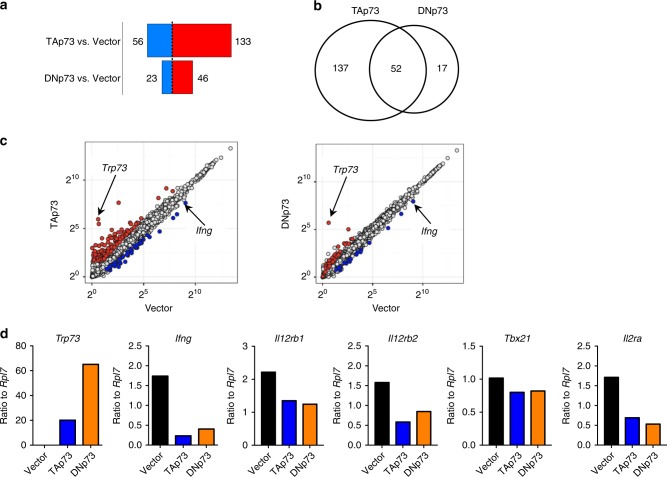
Gene expression analysis of Th1 cells transduced with TAp73 or DNp73 constructs. **a**–**d** Empty vector (Vector), TAp73, or DNp73 were expressed in Th1 cells via retroviral transduction. Transduced cells were identified as GFP^+^ and enriched by cell sorting. mRNA was isolated from GFP^+^ cells and analyzed by RNA-Seq. **a** Number of differentially expressed genes (fold change ≥2 and FDR < 0.05) in TAp73 versus vector and DNp73 versus vector. Upregulated genes are in red and downregulated genes are in blue. **b** Venn diagram showing genes differentially expressed by TAp73 or DNp73 versus vector control, with 52 genes in the intersection area, of which 37 were induced by both TAp73 and DNp73, 14 were repressed by both, and *Lmo2* was inhibited by TAp73 but induced by DNp73. **c** Genes induced and repressed (≥2-fold difference; FDR < 0.05) in TAp73 or DNp73 transduced Th1 cells compared to vector alone. The Log_2_(RPKM + 1) values were plotted and shown as scatter plots. Differentially expressed genes (≥2-fold difference; FDR < 0.05) are shown as open circles in red (higher expression) or blue (lower expression) with TAp73 (left panel) or DNp73 (right panel). **d** Experiments were performed as in **a**–**c**; shown are mRNA expression levels of *Trp73*, *Ifng*, *Il12rb1*, *Il12rb2*, *Tbx21*, and *Il2ra* normalized to *Rpl7* in cells transduced with TAp73 or DNp73. mRNA levels were measured by qRT-PCR from three independent experiments, and representative results are shown. Source data for **d** are provided in a Source Data File.

To elucidate the mechanism underlying p73-mediated inhibition of IFNγ expression, we used RNA-Seq to compare the gene expression profiles of control (vector transduced) Th1 cells with Th1 cells overexpressing TAp73 or DNp73 (Supplementary Data 2). In cells in which TAp73 was overexpressed, 133 genes were upregulated and 56 genes were downregulated (>2-fold; false discovery rate (FDR) < 0.05) (Fig. 3a, Supplementary Data 3), whereas overexpression of DNp73 increased the expression of 46 genes and reduced the expression of 23 genes (Fig. 3a, Supplementary Data 4). Thus TAp73 overexpression seems to have a more potent effect. There were a total of 206 differentially expressed genes. Of these, 52 genes were regulated by both TAp73 and DNp73 overexpression (Fig. 3b), and the other genes were distinctively affected by TAp73 versus DNp73. Corresponding to our RT-PCR results (Fig. 1c), both TAp73 and DNp73 decreased the expression of *Ifng* by RNA-Seq (Fig. 3c, Supplementary Data 2). Analysis of other Th1-related genes revealed that p73 also inhibited *Il12rb1* and *Il12rb2* mRNA expression but had little effect on *Tbx21* mRNA expression; p73 also decreased *Il2ra* mRNA expression (Supplementary Data 2). qRT-PCR analysis confirmed that both TAp73 and DNp73 significantly reduced the expression of *Ifng*, *Il12rb1*, *Il12rb2*, and *Il2ra* but not *Tbx21* mRNA (Fig. 3d).

**Fig. 4:**
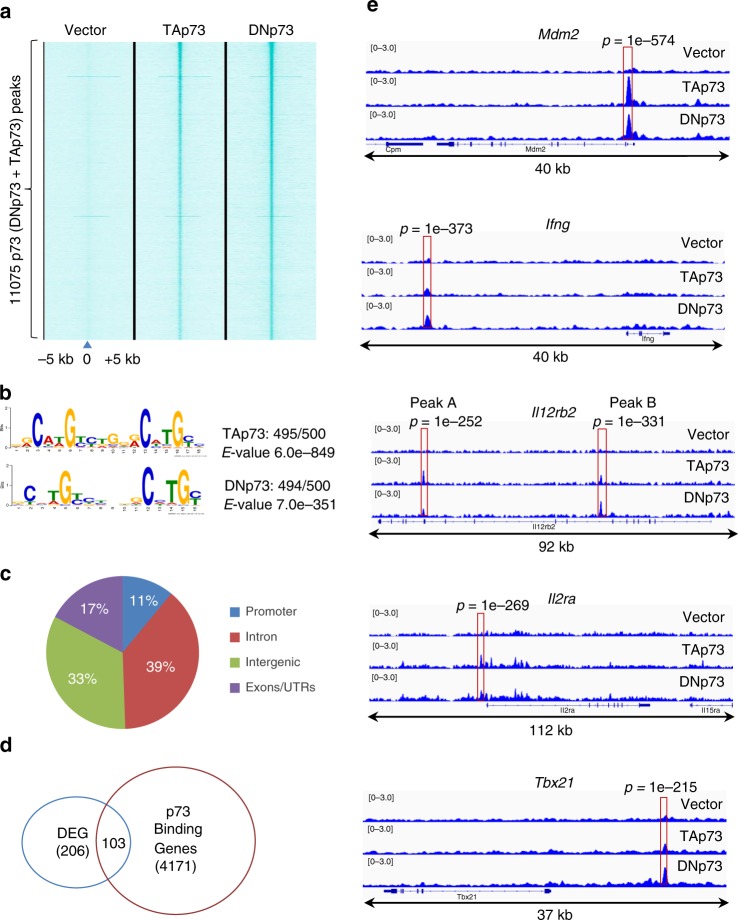
Discovery of p73 direct target genes. **a**–**e** p73-specific binding sites were found via ChIP-Seq analysis from Th1 cells in which FLAG-tagged TAp73 or FLAG-tagged DNp73 was expressed. **a** Each column represents p73 binding in empty vector (EV), FLAG-tagged TAp73, or FLAG-tagged DNp73 within a 10-kb window [−5k, 0, +5k], centered on the p73-binding summits (indicated as position “0”). The intensity of p73-binding peaks is indicated by the intensity of cyan color, whereas “white” represents no binding. **b** The consensus motif for p73 was derived from the top 500 p73-binding summits using MEME. **c** Shown is genome-wide distribution of p73-binding sites at introns, intergenic regions (defined according to RefSeq), promoter regions (15 kb 5′ of the transcription start site), and exons/UTRs regions. **d** Venn diagram of genes differentially expressed upon p73 overexpression and/or directly bound by either TAp73 or DNp73. **e** The gene tracks represent TAp73- and DNp73-binding sites found by ChIP-Seq analysis using anti-FLAG at the *Mdm2*, *Ifng*, *Il12rb2*, *Il2ra*, and *Tbx21* loci. For each peak, the *P* value was calculated using a dynamic Poisson distribution to capture local biases in read background. Three independent ChIP-Seq experiments were performed; representative results are shown.

We next performed chromatin immunoprecipitation–sequencing (ChIP-Seq) analysis on Th1 cells to determine whether p73 could directly bind to the genes identified in the RNA-Seq analysis. Because the p73 antibodies we tested did not work for ChIP-Seq, we instead used an anti-FLAG monoclonal antibody (M2) and Th1 cells in which N-terminally FLAG-tagged TAp73 or DNp73 was transduced. We confirmed that FLAG-TAp73 and FLAG-DNp73 inhibited IFNγ expression, analogous to what we had observed with the native versions of these proteins (Supplementary Fig. 3a), and that both FLAG-TAp73 and FLAG-DNp73 constructs had relatively similar transduction efficiency and expression compared to that of the control vector (Supplementary Fig. 3b, c). We identified a total of 11,075 p73-binding sites using ChIP-Seq analysis, including 4022 TAp73- and 9765 DNp73-binding peaks (Fig. 4a, Supplementary Data 5). Nearly all (>98%) of the top 500 TAp73- or DNp73-binding sites had a canonical p73 recognition motif (Fig. 4b). Thirty-three percent of the p73-binding sites were in intergenic regions, but 67% of the binding sites were in promoter, intron, or exon/untranslated gene body regions (Fig. 4c), and we assigned these gene body p73-binding peaks to the nearest transcription start site, thereby identifying 4171 p73 putative target genes. Fifty percent of the 206 differentially expressed genes identified in the RNA-Seq analysis were bound by p73 (Fig. 4d), suggesting that they may be direct p73 targets. These include *Mdm2*, a known p73 target gene, as well as *Ifng*, *Il12rb2*, and *Il2ra*, which we show can bind p73 (Fig. 4e) and whose expression was regulated by p73 (Fig. 3d). Although *Tbx21* gene expression was not significantly inhibited by p73 (Fig. 3d), we found a p73-binding peak 12 kb upstream from its gene body (Fig. 4e).

**Fig. 5:**
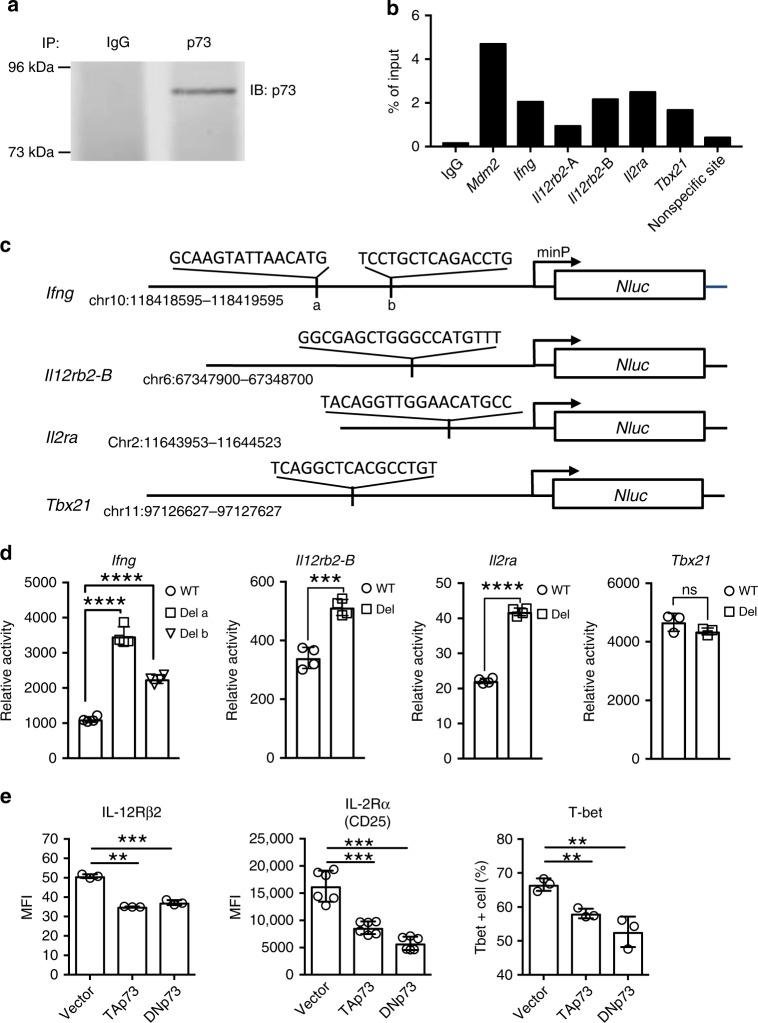
p73 binding regulates target gene expression. **a**, **b** p73 binding at the indicated loci in Th1 cells were assayed by ChIP using anti-p73 antibody versus IgG as a control. p73 protein was immunoprecipitated from Th1 cells and visualized by western blotting with anti-p73 (**a**). A full scan blot image is provided in Supplementary Fig. 9b. The binding was quantified by qPCR (primers to amplify the sites are in Supplementary Table 7; the nonspecific site is from the *Il9* gene). Shown is the percentage of total input (**b**). In **b**, the experiment was performed *n* = 3 times *n* = 2 replicates in each experiment, and representative results are shown. **c** Schematic of the domain structure of WT and p73-binding motif deletion reporter mutations in the *Ifng*, *Il12rb2*, *Il2ra*, and *Tbx21* loci. p73-binding regions from the indicated chromosomal locations were inserted upstream of minimal promoter (minP) and nano-luciferase gene (*Nluc*). WT reporter constructs retained the indicated p73-binding motifs, whereas the constructs with deletion mutants lacked the indicated nucleotide sequences. For *Ifng*, two deletion mutants were generated—one lacking the a motif and the other lacking the b motif. For *Il12rb2*, we tested a deletion mutant corresponding to the peak B motif. **d** The indicated reporter constructs containing WT or p73-binding motif deletion mutants were transfected into Th1 cells. Reporter activity was normalized against co-transfected control luciferase reporter construct activity and shown as relative activity. **e** The level of IL-12Rβ2, IL-2Rα (CD25), and T-bet in Th1 cells expressing control vector, TAp73, or DNp73 was measured by flow cytometric staining and shown as MFI. In **d**, **e**, experiments were repeated *n* = 3 times. In the experiment shown in **d**, the number of replicates was *n* = 4 for *Ifng*, *Il12rb2*-B, and *Il2ra* and *n* = 3 for *Tbx21*. In the experiment shown in **e**, the number of replicates was *n* = 3 for IL-12Rβ2 and T-bet and *n* = 6 for IL-2Rα. Representative results are shown. All data are shown as mean value ± SD and analyzed by a two-tailed unpaired *t* test. The *P* values are indicated (**P < 0.01; ***P < 0.001; ****P < 0.0001). Source data for **a**, **b**, **d**, and **e** are provided in Source Data Files.

Although our p73 antibody did not work well for ChIP-Seq, we were able to use it in ChIP-PCR experiments to confirm endogenous p73 binding at these loci. p73 was indeed immunoprecipitated from WT Th1 cells (Fig. 5a), and p73 binding was significantly enriched at the *Mdm2*, *Ifng*, *Il12rb2* (peaks A and B), *Il2ra*, and *Tbx21* loci but not at a non-specific site (Fig. 5b). To determine whether p73 binding could affect the expression of these genes, we generated WT or p73-binding motif deletion reporter constructs (Fig. 5c; deleted regions are indicated). There are two p73-binding motifs (motifs a and b) near the *Ifng* gene (−22 kb region) (Fig. 5c), and deletion of either motif increased reporter activity (Fig. 5d). Similarly, deletion of the p73-binding motif at peak B in the *Il12rb2* locus or the motif at the *Il2ra* locus (Fig. 4e) also significantly increased reporter activity (Fig. 5d), suggesting a negative regulatory role for p73 on these genes. In contrast, little if any effect was seen when the p73 motif in the *Tbx21* reporter construct was deleted (Fig. 5d), consistent with our not having found a significant effect of p73 on expression of this gene (Fig. 3d). Analogous to the repression of *Ifng* expression by p73 in Th1 cells (Fig. 1c), expression of interleukin (IL)-12Rβ2 and IL-2Rα (CD25) were also significantly reduced when p73 was overexpressed (Fig. 5e) although the effect on the percentage of T-bet^+^ cells was relatively modest (Fig. 5e). Thus there are multiple potential p73 target genes that might affect Th1 differentiation.

### Trp73 gene deletion reduces disease severity in EAE mice

**Fig. 6:**
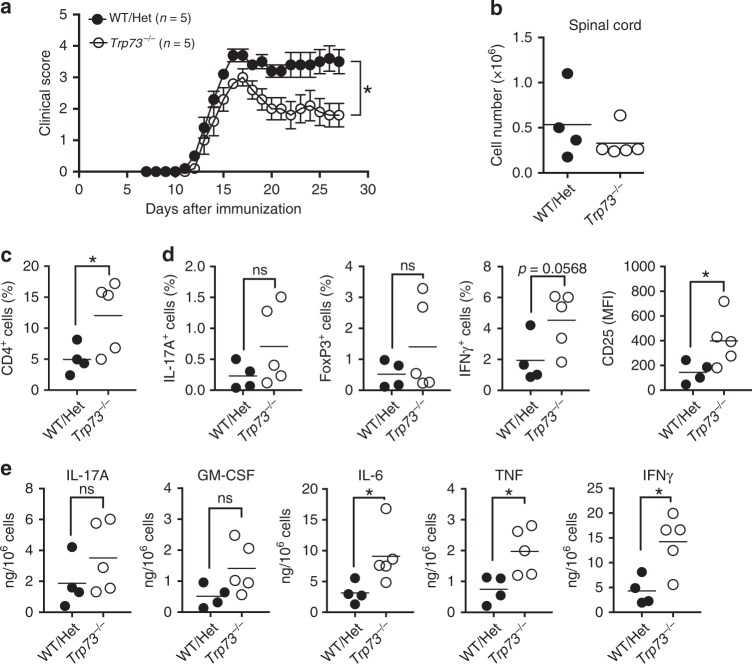
*Trp73* gene deletion reduces disease severity in EAE mice. **a** Clinical scores for groups of *Trp73*^−/−^ mice and wild-type/heterozygous (WT/Het) littermates after immunization with MOG_35–55_ are shown as mean values ± S.E.M. Data were analyzed by a two-tailed unpaired *t* test; *P* values are indicated (*P < 0.05). **b**–**e** Total lymphocytes were isolated from the spinal cords from WT/Het (*n* = 4) or *Trp73*^−/−^ (*n* = 5) mice 27 days after EAE induction. **b** Total recovered cell numbers. **c** Infiltrating lymphocytes from the spinal cord were analyzed by flow cytometry and the percentage of CD4^+^ T cells is shown. **d**, **e** Total cells from the spinal cord were stimulated with MOG_35–55_ for 48 h, and then the percentage of IL-17A^+^CD4^+^, FoxP3^+^CD4^+^, and IFNγ^+^CD4^+^ cells and CD25 expression (MFI) were determined by flow cytometry and shown as indicated. **e** IL-17A, GM-CSF, IL-6, TNF, and IFNγ levels in culture supernatant after MOG_35–55_ stimulation were determined using a LEGENDplex Multi-Analyte assay. The results were normalized against total cell numbers. All data are presented as scatter dot plots with mean values indicated and analyzed by a two-tailed unpaired *t* test using mean ± SEM values; *P* values are indicated (ns *P* ≥ 0.05; *P < 0.05). Data are from one representative of three experiments with *n* = 5 mice per group in each experiment. Source data for **a**–**e** are provided in a Source Data File.

The above data demonstrated that p73 negatively regulates IFNγ production in vitro. To investigate the importance of this repressive effect in vivo, we used experimental autoimmune encephalomyelitis (EAE), a mouse model of MS in which disease severity is mainly driven by Th1 and Th17 cells^25^. We used *Trp73*^−/−^ mice, which have previously been reported to have relatively normal T cell development^16,26,27^, and indeed basic phenotypic profiling of *Trp73*^−/−^ mouse thymus and spleen revealed only a small increase in the percentage of double positive thymocytes, with similar overall T cell populations to WT mice (Supplementary Fig. 4). *Trp73*^−/−^ mice and WT or heterozygous (Het) littermates were immunized with MOG_35–55_ to induce EAE, and disease severity was monitored for 27 days. While there was no significant difference in the time required for disease onset, *Trp73*^−/−^ mice had significantly reduced disease severity (*P* < 0.05) and better recovery than was observed in littermate controls (Fig. 6a). We next examined the immune cells present in spinal cord at the end of the experiment (day 27) and found that there tended to be fewer infiltrating lymphocytes in the *Trp73*^−/−^ mouse spinal cords (Fig. 6b) than in heterozygous *Trp73*^*+/−*^ or WT mice, but there was an increase in the percentage of CD4^+^ cells in the spinal cords of *Trp73*^−/−^ mice (Fig. 6c). In contrast, there was no significant difference in the cellularity of draining lymph nodes (dLNs; Supplementary Fig. 5a) nor in the percentages of CD4^+^ T cells (Supplementary Fig. 5b) or IFNγ, IL-17A, and FoxP3 producing CD4^+^ T cells in the dLNs (Supplementary Fig. 5c). In the spinal cord, however, after MOG_35–55_ stimulation, although not statistically significant, there was a possible trend toward an increase in the IL-17A^+^ and FoxP3^+^ CD4^+^ T cells infiltrating the spinal cord in *Trp73*^−/−^ mice, a nearly significant increase in the percentage of IFNγ-producing CD4^+^ cells, and increased CD25 expression (Fig. 6d), consistent with p73 negatively regulating *Il2ra* expression (Figs. 3e and 5e). There tended to be more IL-17A and granulocyte macrophages colony-stimulating factor and there was an increase in IL-6, tumor necrosis factor, and IFNγ in the supernatant of the infiltrating cells from *Trp73*^−/−^ mice (Fig. 6e). Thus, in the context of EAE, *Trp73*^−/−^ mice had a lower clinical score and higher IFNγ production by T cells infiltrating into the spinal cord.

### Trp73 deletion enhances IFNγ response in a colitis model

**Fig. 7:**
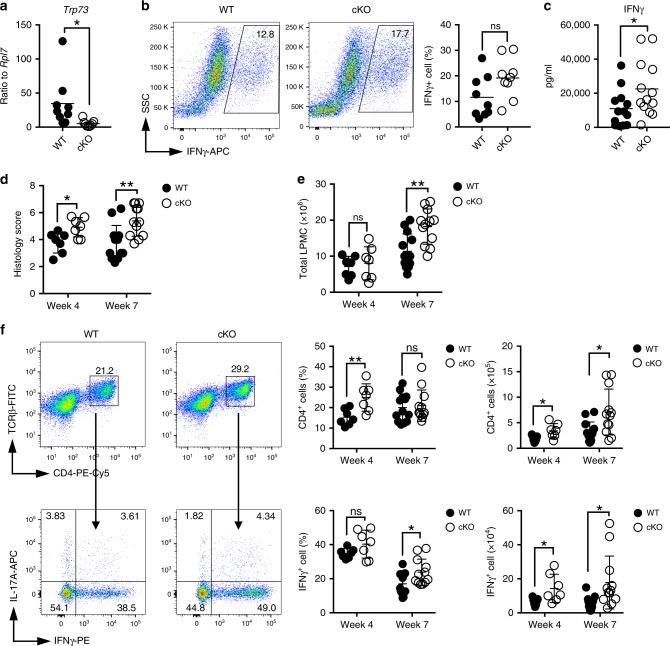
Augmented IFNγ production following in vitro differentiation and enhanced pathogenic IFNγ-producing T cell induction and disease mediated by *Trp73*-deficient T cells in an adoptive T cell transfer model of colitis. **a**–**c** Naive CD4^+^ cells from WT and *Trp73* conditional knockout (cKO) mice were differentiated into Th1 cells for 2 days. **a** mRNA expression of *Trp73* were assessed by qRT-PCR. Data are pooled results from two independent experiments with *n* = 9 mice per group in total. **b** Intracellular IFNγ levels were measured by flow cytometric staining; a representative FACS plot is shown on the left and summarized IFNγ expression data are shown on the right. **c** Secreted IFNγ levels (right panel) were measured by LEGENDplex^TM^ assay. Data for **b**, **c** are pooled results from three independent experiments with *n* = 13 mice per group in total. All data from **a**–**c** are presented as scatter dot plots with mean value indicated and *P* values were calculated using mean ± SEM by two-tailed unpaired Student’s *t* test. **d**–**f** CD4^+^CD45RB^hi^ T cells from WT or *Trp73* cKO mice were adoptively transferred into *Rag2*^−/−^ recipients to induce colitis. Colonic histology scores at weeks 4 and 7 post-transfer are shown in **d**. Total colonic lamina propria mononuclear cell (LPMC) counts from the indicated time points are shown in **e**. **f** Infiltrating CD4^+^ T cells in the colon were measured by flow cytometry and the percentage and number of CD4^+^ T cells are shown. IFNγ-producing CD4^+^ T cells in the colon were determined by intracellular staining, and the percentage and number of IFNγ^+^ CD4^+^ T cells are indicated. Data for **d**–**f** are combined from two experiments with 9–10 mice per group in each experiment. In total, *n* = 7 mice per group were analyzed at week 4, and *n* = 13 (WT) and *n* = 12 (*Trp73* cKO) mice per group were analyzed at week 7. All data from **d**–**f** are presented as scatter dot plots with mean ± SD indicated and analyzed by two-tailed unpaired Student’s *t* test with Welch’s correction, and the *P* values are indicated (ns *P* ≥ 0.05; *P < 0.05; **P < 0.01). Source data for **a**–**f** are provided in Source Data Files.

To further investigate the role of *Trp73* in a different T cell-mediated disease model, we crossed *Trp73*^fl/fl^ mice to CD4^Cre^ mice to delete *Trp73* in CD4^+^ T cells (*Trp73* cKO). Like the complete *Trp73*^−/−^ mice, there was no significant effect on thymic or splenic lymphoid development in these animals (Supplementary Fig. 6). We therefore compared Th1 differentiation in WT versus *Trp73* cKO T cells. Upon in vitro differentiation in the presence of IL-12 and anti-IL-4, we confirmed that *Trp73* mRNA expression was indeed significantly lower in *Trp73* cKO than in WT CD4^+^ T cells (Fig. 7a), and while the percentage of IFNγ-positive cells measured by intracellular staining tended to be higher in *Trp73* cKO T cells but did not reach statistical significance (Fig. 7b), *Trp73* cKO T cells secreted significantly higher levels of IFNγ compared to WT cells (Fig. 7c). We therefore further analyzed the impact of *Trp73* deletion in vivo by assessing disease development in a T cell adoptive transfer model of experimental colitis that is characterized by intestinal inflammation driven by an unrestrained Th1/Th17 CD4^+^ T cell response to commensal microbial, and possibly self-antigens, resembling human Crohn’s disease^28,29^. In this model, transfer of naive CD4^+^CD45RB^hi^ T cells from WT mice into *Rag2*^−/−^ mice results in frank colitis development 8–10 weeks after transfer in our animal facilities. Transfer of CD4^+^CD45RB^hi^ T cells from *Trp73* cKO mice into *Rag2*^−/−^ mice resulted in accelerated colitis development compared to mice that received WT CD4^+^CD45RB^hi^ T cells from littermate controls. As early as 4 weeks after T cell transfer, mice transferred *Trp73*-KO T cells had more inflamed colons by histology (Fig. 7d). Although there was no significant change in total lamina propria mononuclear cell (LPMC) numbers at this early time point (Fig. 7e), a higher accumulation of total as well as pathogenic IFNγ-producing CD4^+^ T cells in the colon was evident (Fig. 7f). Moreover, although total IL-17A^+^ CD4^+^ T cells were not increased, there were increased IFNγ^+^IL-17A^+^ double-producing CD4^+^ T cells (Supplementary Fig. 7a). Furthermore, at week 7 post T cell transfer, while there was no significant difference in body weights, which is not unexpected at this still early time point in disease development (Supplementary Fig. 7b), *Rag2*^−/−^ mice receiving *Trp73* cKO T cells developed symptoms of colitis including diarrhea, as compared to mice receiving T cells from WT littermates, which displayed no symptoms of colitis. The *Rag2*^−/−^ mice receiving *Trp73* cKO T cells also had increased tissue inflammation reflected in higher histology scores (Fig. 7d), total numbers of LPMC (Fig. 7e), and IFNγ-producing CD4^+^ T cells (Fig. 7f). Together, these data demonstrate an accelerated disease course and increased colitis severity following adoptive transfer of *Trp73* cKO compared to WT CD4^+^CD45RB^hi^ T cells to *Rag2*^−/−^ recipients, and are consistent with the ability of *Trp73* to restrain pathogenic Th1 differentiation, particularly at an early stage in disease development.

## Discussion

T helper cell differentiation is programmed by master regulators and is further fine-tuned by a complex gene network that is not yet fully understood. Using HBCGM analysis and cells from 16 strains of mice, we identified transcription factor p73 as a potential regulator of Th1 differentiation. Here we have provided multiple lines of evidence that p73 negatively regulates IFNγ production by Th1 cells. Interestingly, our data indicate that p73 expression is very low in naive CD4^+^ T cells and that its expression is upregulated in differentiated Th1. This expression pattern is consistent with the possibility that it acts as part of a negative feedback loop that regulates Th1 differentiation, which is consistent with the results obtained in the shRNA experiments.

Interestingly, it has been reported that p73 directly interacts with STAT1^22^. Our results indicate that, although *Ifng* expression was lower in the absence of STAT1, p73 could still decrease *Ifng* expression, with similar findings in the absence of STAT4. In fact, we show direct binding of p73 to the *Ifng* gene and that mutation of this p73 site results in augmented activity of a reporter construct containing the site, with analogous results for *Il12rb2*, which also is important for Th1 differentiation. This suggests potential direct inhibitory effects of p73 on the expression of these genes. Interestingly, p73 also bound to the *Il2ra* gene (encoding the IL-2 receptor α chain) and its expression was also inhibited by p73. Because IL-2 can augment the expression of IL-12Rβ1 and IL-12Rβ2^30,31^, the inhibition of IL-2Rα by p73 might inhibit IL-2-induced IL-12 receptor expression and thus Th1 differentiation^32^, providing another mechanism for the effect of p73 on *Ifng* expression.

In EAE, we found that IFNγ expression was increased in the absence of p73, consistent with the ability of p73 to inhibit IFNγ expression. Interestingly, however, disease severity was reduced. While IFNγ plays a pathogenic role during the induction phase of EAE, accumulating evidence indicates that IFNγ production plays a protective role during the chronic phase of MS in humans and EAE in mice^33^. The improved recovery of *Trp73*^−/−^ mice from EAE and their elevated production of IFNγ by spinal cord infiltrating cells are consistent with IFNγ playing a protective role during the chronic phase of this disease. However, in the T cell transfer model of inflammatory bowel disease, we found accelerated and more severe colitis in *Rag2*^−/−^ mice transferred CD4^+^CD45RB^hi^ naive T cells lacking *Trp*73 compared to cells from WT littermates, together with the early and sustained development of pathogenic Th1 T cells in the colon tissues, consistent with the ability of *Trp*73 to restrain IFNγ production and Th1 development in a T cell-intrinsic manner.

Our data thus define a novel function for p73 in immune regulation, which affects the IFNγ response and is relevant to autoimmune disease. While p73 has not yet been reported to have a role in a human inflammatory or autoimmune disease, there is a human single-nucleotide polymorphism (SNP) within a *TP73* intron (dbSNP: rs12027041) with modest association (*P* = 1.1E−5) to rheumatoid arthritis based on the HGVST10 GWAS database^34^. Thus our identification of p73 as a negative regulator of Th1 differentiation based on higher IFNγ production may help to elucidate the mechanism(s) underlying inter-individual differences in immune responses that contribute to disease susceptibility. Moreover, because abnormal p73 expression is highly correlated with tumor grade and clinical outcome, especially in hematological malignancies^15^, it is possible that altered p73 expression might affect tumor development and progression based on its modulation of IFNγ production. Furthermore, the discovery of a role for p73 in Th cells suggests possible crosstalk between inflammatory responses and tumorigenesis, with possible therapeutic ramifications.

## Methods

### Mouse strains

Different inbred strains of mice (C57BL/6J, 129S1/SvImJ, A/J, AKR/J, C3H/HeJ, DBA/2J, NOD/LtJ, BALB/cJ, CBA/J, LP/J, SJL/J, MRL/MpJ, NZB/BlnJ, NZW/LacJ, SM/J, FVB/NJ), *Stat1*^*−/+*^ (*Stat1*^tm1Dlv^), and *Stat4*^*−/+*^ (*Stat4*^*em3Adiuj*^) mice were purchased from the Jackson Laboratory. The stock numbers for these mouse strains are provided in Supplementary Table 2. *Trp73*^−/−^ (*Trp73*^*tm1a(KOMP)Wtsi*^) embryonic stem (ES) cells were purchased from the knockout consortium. The details of knockout strategy can be found at KOMP repository website (http://www.Komp.org) with the project ID: CSD89710. *Trp73*^−/−^ mice were then generated from ES cells in the NHLBI transgenic core facility. *Trp73*^f/f^ floxed mice (exon 5 was flanked with *loxP* sites) were generated by Flp recombination from *Trp73*^−/−^ mice. Then *Trp73* conditional knockout mice (*Trp73* cKO) were generated by crossing *Trp73*^f/f^ mice with CD4-*Cre* mice. All *Trp73*^−/−^ and *Trp73* cKO were further backcrossed at least four generations to the C57BL/6 background. All mice were co-housed in a specific pathogen-free animal facility and euthanized by carbon dioxide inhalation. Animal protocols were approved by the NHLBI Animal Care and Use Committee and followed the NIH Guidelines “Using Animals in Intramural Research”.

### Haplotype-based computational genetic mapping

Genetic factors were identified using the HBCGM methods^17,35^^,^. In brief, HBCGM utilizes bi-allelic SNPs that are polymorphic among the analyzed strains. These SNPs usually display a limited degree of variation locally among the strains. Haplotype blocks were constructed, and the pattern of genetic variation within each block was correlated with the distribution of trait values among the strains analyzed by using analysis of variance (ANOVA)-based statistical modeling^18,35^. *P* values from the ANOVA model and the corresponding genetic effect size were calculated for each block. The blocks were then ranked by their *P* values, and those below an input threshold were used as candidate predictions.

### Mouse *Trp73* constructs

A cDNA clone corresponding to mouse DNp73 (accession number: BC066045.1) was purchased from Mammalian Gene Collection. The cDNA fragment corresponding to the coding region of mouse TAp73 (accession number: NM_011642) was synthesized by Eurofins Genomics and cloned into pBluescriptSKII by restriction enzyme cutting. The cDNA encoding mouse p53 (accession number: NM_011640) was obtained by PCR from a cDNA pool of CD4^+^ T cells from C57BL/6J mice and then cloned into pBluescriptSKII. All cDNA constructs were verified by sequencing and then subcloned into retroviral expression vector pRV-GFP, which contains an IRES element 5’ of the green fluorescent protein (GFP) gene. All p73 mutants were generated by PCR and verified by sequencing.

### Th1 polarization

Naive CD4^+^ T cells were purified from the spleens of 7–8-week-old female mice using a CD4^+^CD62^+^ T cell Isolation Kit II (Miltenyi). Mouse naive CD4^+^ T cells were cultured at 10^6^/ml in RPMI 1640 medium containing 10 mM Hepes, 10% fetal bovine serum, 2 mM L-glutamine, and antibiotics, including 50 μM 2-ME, and polarized under Th1 conditions for 3 days with plate-bound anti-CD3 (2 µg/ml), soluble anti-CD28 (1 µg/ml, PharMingen), 10 ng/ml mouse IL-12 (PeproTech), and 10 μg/ml of anti-mIL-4 antibody^36^.

### Quantitative RT-PCR

RT-PCR was performed by standard methods according to the manufacturer’s instructions. Primers and probes were from ABI. Data were collected and quantified using the CFX Manager v3.1 (BioRad) software. Expression levels were normalized to *Rpl7*^36^.

### Cell staining and flow cytometric analyses

Cells were stained with surface marker antibodies in fluorescence-activated cell sorting (FACS) buffer (phosphate-buffered saline (PBS) + 0.5% bovine serum albumin (BSA)), PerCP/Cy5.5 anti-CD25 (Biolegend, Clone PC61) or phycoerythrin anti-IL12Rb2 (Miltenyi Biotec, Clone REA200). For intracellular staining, cells were fixed and permeabilized by Cytofix/Cytoperm Buffer (BD Biosciences) after they were re-stimulated with 100 nM of phorbol myristate acetate (PMA), 500 ng/ml of ionomycin, and BD GolgiPlug (BD Bioscience) for 4 h. Cells were then stained with isotype control antibodies or allophycocyanin anti-IFNγ (Biolegend, CloneXMG1.2) or BV421 anti-T-bet (Biolegend, Clone 4B10). Stained cells were analyzed on a LSRFortessa^TM^ flow cytometer (Becton Dickinson) using BD FACS Diva v8.0.1 for data collection and FlowJo software v10 (Tree Star, Inc.) for analysis.

### Retroviral transduction experiments

cDNAs corresponding to different *Trp73* isoforms and truncated mutants were cloned into pRV, a GFP-expressing retroviral vector, and transfected with the pCl-Eco packaging plasmid into 293T cells. Retroviral supernatant was mixed with 8 μg/ml polybrene and virus was introduced by centrifugation at 3500 rpm for 45 min at 30 °C into mouse CD4^+^CD62L^+^ T cells, which had been pre-activated under Th1 differentiation conditions. The supernatant was replaced with new medium, and cells were cultured as indicated for 3 days and then harvested for intracellular cytokine staining or transduced cells (GFP^+^) were sorted by FACS and harvested for RNA preparation.

### Retroviral *Trp73*-shRNA knockdown

shRNAs to *Trp73* in a retroviral vector pSIREN-RetroQ-ZsGreen1 were purchased from Clontech Laboratories and transfected into 293T cells with packing plasmid (pCl-Eco). Retrovirus in the supernatant was introduced into mouse CD4^+^CD62L^+^ T cells that had been pre-activated under Th1 differentiation conditions by spin infection. Supernatant was replaced with new medium; cells were cultured under Th1 conditions for 3 days and then assayed with intracellular cytokine staining; or transduced cells (GFP^+^) were sorted out by FACS and harvested for RNA preparation.

### Western blotting

Whole-cell lysates were prepared with RIPA buffer, then Protein Sample Loading Buffer (Li-COR Biosciences) was added. Samples were heated for 5 min at 95 °C, loaded onto NuPAGE Bis-Tris Gels for sodium dodecyl sulfate-polyacrylamide gel electrophoresis (ThermoFisher), and then transferred onto Immobilon-FL PVDF Membranes (Millipore Sigma). Membranes were blocked in PBS containing 5% BSA and then incubated with primary antibody and fluorescent-conjugated secondary antibodies. The image data were acquired with an Odyssey CLx system (LI-COR) and processed with the ImageStudio Lite (LI-COR) software.

### RNA-Seq analysis

*Trp73*-overexpressed Th1 cells were isolated by FACS sorting as GFP^+^ cells 3 days after retroviral transduction, and total RNA was then extracted. RNA-Seq libraries were prepared from mRNA (isolated from 2 μg total RNA) using the KAPA Kit (Kapa Biosystems, Wilmington, MA) per the manufacturer’s protocol. PCR products were “barcoded” (indexed) and sequenced on an Illumina Hi-Seq 2000. To analyze RNA-Seq data, sequenced reads (50 bp, single end) were mapped to the mouse genome (mm10, December 2011 Assembly) using Tophat v2.2.1, and the gene expression was calculated using RSEM v1.2 with the following parameters (–forward-prob 0–bowtie-n 1–bowtie-m 100–seed-length 28)^36,37^. The differentially expressed genes were identified using edgeR^38^ and visualized using R packages (gglot2 v3.2.1 and pheatmap v1.0.12). The negative binomial distribution was used to calculate the exact *P* values for differential expression, and FDR was calculated using the Benjamini–Hochberg correction.

### ChIP-Seq and ChIP analysis

Th1 cells transduced with either the vector control or *Trp73* were cross-linked with 1% formaldehyde (methanol-free, Pierce, Rockford, IL) at room temperature for 10 min. They were then subjected to sonication and fragmented chromatin equivalent to 10 million cells was immunoprecipitated using 5 μg of normal mouse IgG as a control or anti-FLAG (M2) antibody (Sigma) and Magna ChIP^TM^ Protein A+G Magnetic Beads (Millipore, Billerica MA). ChIP-Seq DNA libraries were generated using the KAPA LTP Library Preparation Kit (Kapa Biosystems, Wilmington, MA) and indexed using primers (BIOO Scientific, Austin, TX). Libraries were then sequenced using an Illumina HiSeq 3000 platform. All sequenced reads (50 bp, single end) were mapped to the mouse genome (mm10, December 2011 Assembly) using bowtie v.1.0.1 using parameter (-m 1)^39^. The peaks were identified using MACS v1.4^40^. For each peak, the *P* value was calculated using a dynamic Poisson distribution to capture local biases in read background levels, and FDR values were calculated using the Benjamini–Hochberg correction. The mapped reads were converted to the tiled data file format using igvtools (–z 5 –w 5 –e 100 –minMapQuality 30) and displayed as custom tracks on the IGV genome browser^41^. The peaks were annotated using the HOMER software^42^. All ChIP-Seq and RNA-Seq samples were sequenced in the NHLBI Sequencing core. ChIP-qPCR from WT Th1 cells using anti-p73 antibody (EP436Y, Abcam) was performed in a similar way as described above and analyzed by qPCR using the primers listed (Supplementary Table 3).

### p73-binding site reporter assay

PCR-generated p73-binding region (~1 kb) fragments were cloned into pNL3.1 (Promega) 5’ of the Nanoluc luciferase gene to generate reporter constructs (Fig. 5c). Both WT constructs or deletion mutants lacking the p73 motifs indicated in Fig. 5c were generated. For each reporter construct, 0.8 μg of the reporter plasmid was mixed with 0.2 μg of control reporter pGL4.54 (Promega) plasmid and then co-transfected into 2 × 10^6^ mouse CD4^+^ T cells that had been cultured under Th1 conditions for 2 days by using the P3 Primary Cell 4D-Nucleofector X Kit (Lonza, V4XP-3032) according to the Amaxa 4D-Nucleofector protocol for mouse T cells (Lonza). Post-transfection, cells were cultured overnight under Th1 conditions and analyzed for Nanoluc luciferase activity using Nano-Glo® Dual-luciferase® reporter assay system (Promega) according to the manufacturer’s instructions.

### EAE induction with MOG_35–55_

Ten-to-12-week-old female and male *Trp73*^−/−^ mice and WT/Het littermates were immunized subcutaneously with MOG_35–55_ peptide in emulsion with complete Freund’s adjuvant (Hooke Laboratories), and injected intraperitoneally (i.p.) with 200 ng of pertussis toxin (Hooke Laboratories) 2 and 24 h after immunization. Animals were monitored daily and clinically scored in a blinded fashion as follows: grade 1, limp tail or isolated weakness of gait without limp tail; grade 2, limp tail and weakness of hind legs or poor balance; grade 3, total hind leg paralysis or severe head tilting; grade 4, total hind leg and partial front leg paralysis; and grade 5, moribund or dead animal.

### Adoptive T cell transfer colitis experiments

Total CD4^+^ splenic T cells were isolated from 6- to 8-week-old female *Trp73* cKO and WT littermates via negative selection using the MACS CD4 T-cell Isolation Kit (Miltenyi Biotec Inc. USA, Auburn, CA). Naive (CD4^+^CD25^−^CD45RB^hi^) T cells were subsequently sorted using a FACSAria^TM^ cell sorter (BD Biosciences, San Jose, CA), and gating strategy for sorting is provided in Supplementary Fig. 8f. In all, 3 × 10^5^ naive T cells were transferred i.p. to 6–8-week-old female C57BL/6 *Rag2*^−/−^ recipient mice (purchased from Taconic Biosciences). Development of intestinal inflammation was monitored by weight loss, which correlates with histological evidence of colitis. Two time points were used to assess the colitis development: 4 weeks after T cell transfer to detect early colitis development and at 7 weeks after T cell transfer to assess colitis severity, when ~20% weight loss (from initial body weight) or other symptoms of colitis such as diarrhea were reported in some but not all mice. Proximal, middle, and distal sections of mouse colons were fixed in 10% formalin, stained with hematoxylin and eosin, and scored blindly for evidence of inflammation according to well-established criteria that include grading for lamina propria cellularity/inflammatory infiltrates (0–3), epithelial hyperplasia and goblet cell depletion (0–3), percentage of tissue involvement (0–3), and markers of severe inflammation including submucosal inflammation and the presence and numbers of crypt abscesses (0–3)^43,44^. LPMCs were isolated from colon tissue by established techniques^44^. Briefly, colon tissue was rinsed with Hank’s Balanced Salt Solution (HBSS) to remove feces and debris, followed by removal of epithelial cells by incubation and vigorous shaking for 15 min at 37 ° C in HBSS supplemented with 15 mM HEPES, 5 mM EDTA (Invitrogen, Carlsbad, CA), 10% fetal calf serum (Gemini Bio-Products, West Sacramento, CA), and 1 mM dithiothreitol (Sigma-Aldrich, St. Louis, MO). After rinsing to remove epithelial cells, the remaining tissue was digested in complete Iscove’s modified Dulbecco’s modified Eagle’s medium containing 0.17 mg/ml liberase TL and 30 μg/ml DNase I (both from Roche Applied Science, Indianapolis, IN) for 1 h at 37 ° C. Single-cell suspensions were made by passing digested tissue through 100- and 40-μm strainers (BD Biosciences, San Jose, CA). LPMCs were collected from individual mice, counted, and analyzed by flow cytometry for the percentage of CD4^+^ T cells and for T cell cytokine production by intracellular staining following PMA/ionomycin stimulation, as described above. Antibodies to CD4, CD25, CD45RB, TCRβ, IFNγ, and IL-17A were from ThermoFisher-eBioscience.

### Statistical analysis

Two-tailed paired *t* tests were performed using Prism 7 (GraphPad software) to evaluate whether the difference between the means of two groups was statistically significant. To compare inter-strain difference versus intra-strain difference, an ANOVA model was applied evaluating the strain-specific effect (SAS, Version 9.4, Cary, NC). The correlation of IFNγ production levels at different time points were evaluated using Spearman’s correlation coefficient using the R software (www.r-project.org).

### Reporting summary

Further information on research design is available in the Nature Research Reporting Summary linked to this article.

## Supplementary information

Supplementary Information

Reporting Summary

Description of Additional Supplementary Files

Supplementary Data 1

Supplementary Data 2

Supplementary Data 3

Supplementary Data 4

Supplementary Data 5

## Data Availability

All relevant data are available from the authors. ChIP-Seq and RNA-Seq data sets have been deposited in the Gene Expression Omnibus under accession number GSE144496. All other source data have been provided in a Source Data File (“Source Data Files.zip”). All the statistics source data for Figs. 1, 2b, 3d, 5a, b, d, e, 6, and 7 and Supplementary Figs. 1, 2b, d, 3b, 4, 5, 6, and 7 are provided as Statistic Source Data as an Excel (xlsx) file. The original flow cytometric data for Figs. 1a and 7b, f and Supplementary Figs. 2a, c and 3a are provided in corresponding folders as Flow Cytometry Standard (FCS) files and gating strategies are provided in Supplementary Fig. 8. Unprocessed western images for Figs. 1e and 5a and Supplementary Fig. 3c are provided in Supplementary Fig. 9.

## Source data

Source Data
