# Supplementary material for: Transcription factor p73 regulates Th1 differentiation
Source: Nat Commun. 2020 Mar 19;11:1475. doi: 10.1038/s41467-020-15172-5 (PMC7081339; doi:10.1038/s41467-020-15172-5)
Supplement: Supplementary file 1 — Supplementary Information [file 41467_2020_15172_MOESM1_ESM.pdf]

Transcription Factor p73 Negatively Regulates Th1 Differentiation

Ren et al.

# Supplementary Figure 1

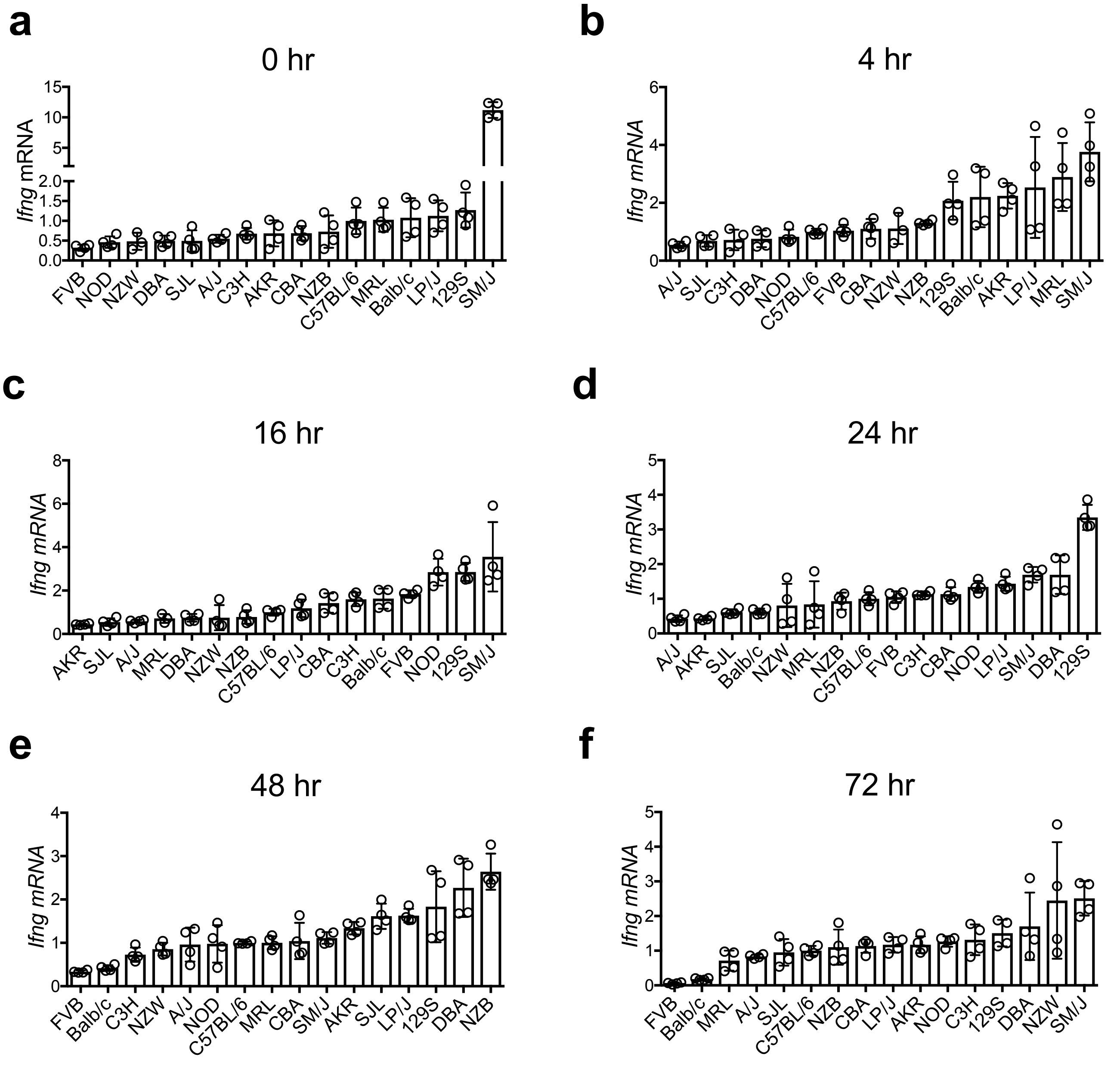


**Supplementary Fig. 1: *Ifng* mRNA expression profiles from 16 inbred mice strains. a-f,** Normalized *Ifng* mRNA expression profiles determined by qRT-PCR of *in vitro* polarized Th1 cells from 16 inbred mice strains at the indicated time points (**a-f** show data for 0, 4, 16, 24, 48, and 72 hr, respectively). Data were pooled measurements (n = 4) from two experiments with two biological replicates (2-5 mice) for each mouse strain in each experiment. The *Ifng* levels from C57BL/6J mice were set at 1.0 and used normalization. Data are presented as mean ± SD. The strains were ordered from lowest to highest *Ifng* mRNA levels at each indicated time point. Source data for **a-f** are provided in a Source Data File.

# Supplementary Figure 2

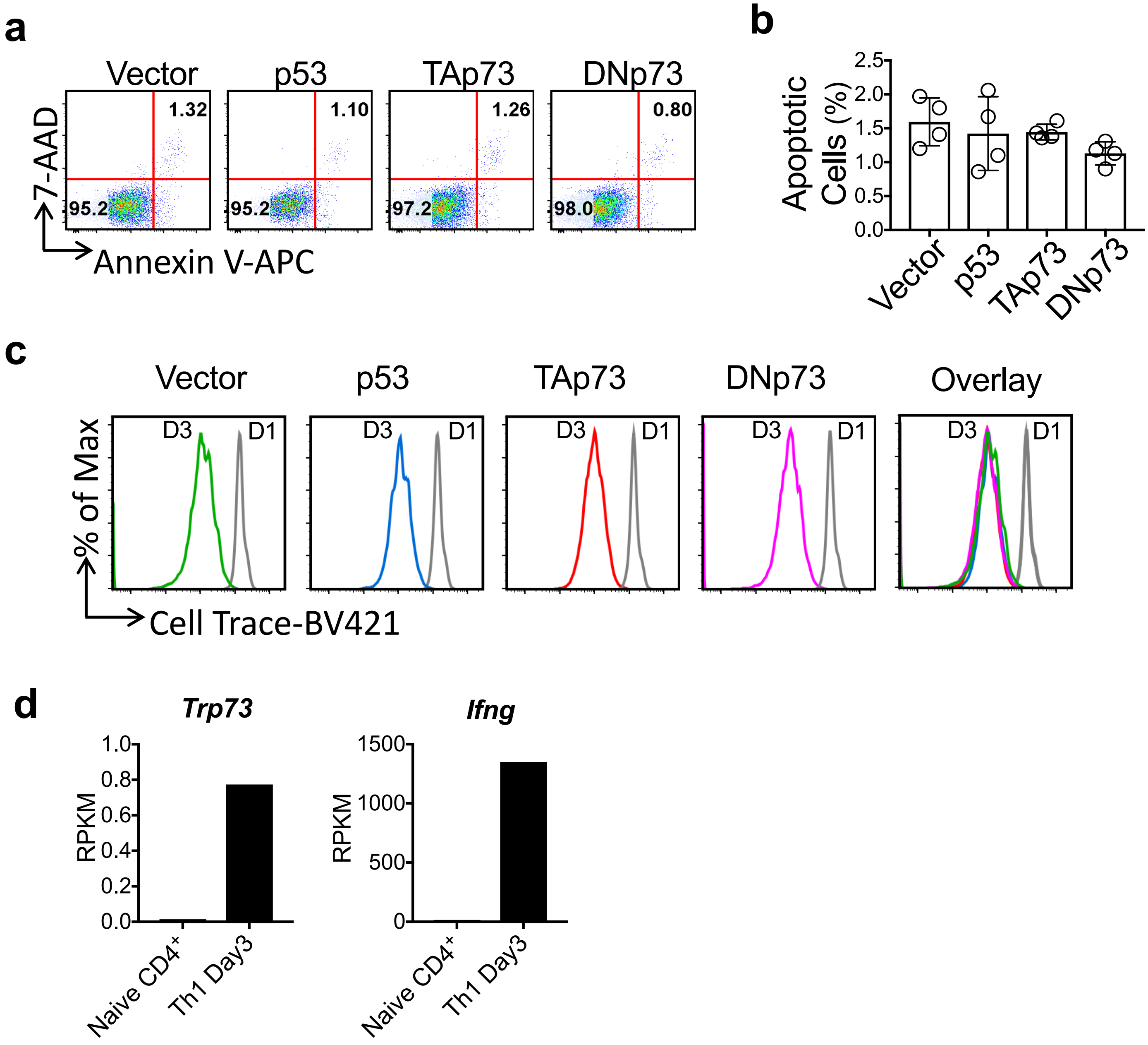


**Supplementary Fig. 2: Overexpression of p73 did not substantially affect Th1 cell survival and proliferation. a-c,** Empty vector, p53, TAp73, and DNp73 were expressed in Th1 cells via retroviral transduction. Th1 cells were transduced with the indicated constructs. **a-b,** The apoptosis level of transduced cells ($GFP^+$) was determined by 7-AAD and annexin V staining followed by flow cytometric analysis (**a**). The results from 4 replicates are shown as mean ± SD (**b**). The experiment was repeated three times and representative data are shown. **c,** The proliferation rate of transduced cells was measured by CellTrace dye dilution assay. The individual and overlay histograms of CellTrace from $GFP^+$ cells from indicated constructs are shown as follows: Day 1 (D1) traces are in grey; for Day 3 (D3) traces, empty vector is green; p53 is blue, TAp73 is red, and DNp73 is magenta. Three independent experiments were performed; representative results are shown. **d**, Naïve $CD4^+$ T cells were isolated from WT C57BL/6 mice and differentiated into Th1 for 3 days. Relative expression level of *Trp73* and *Ifng* was determined by RNA-Seq; shown is RPKM. Source data for **a-d** are provided in Source Data Files.

# Supplementary Figure 3

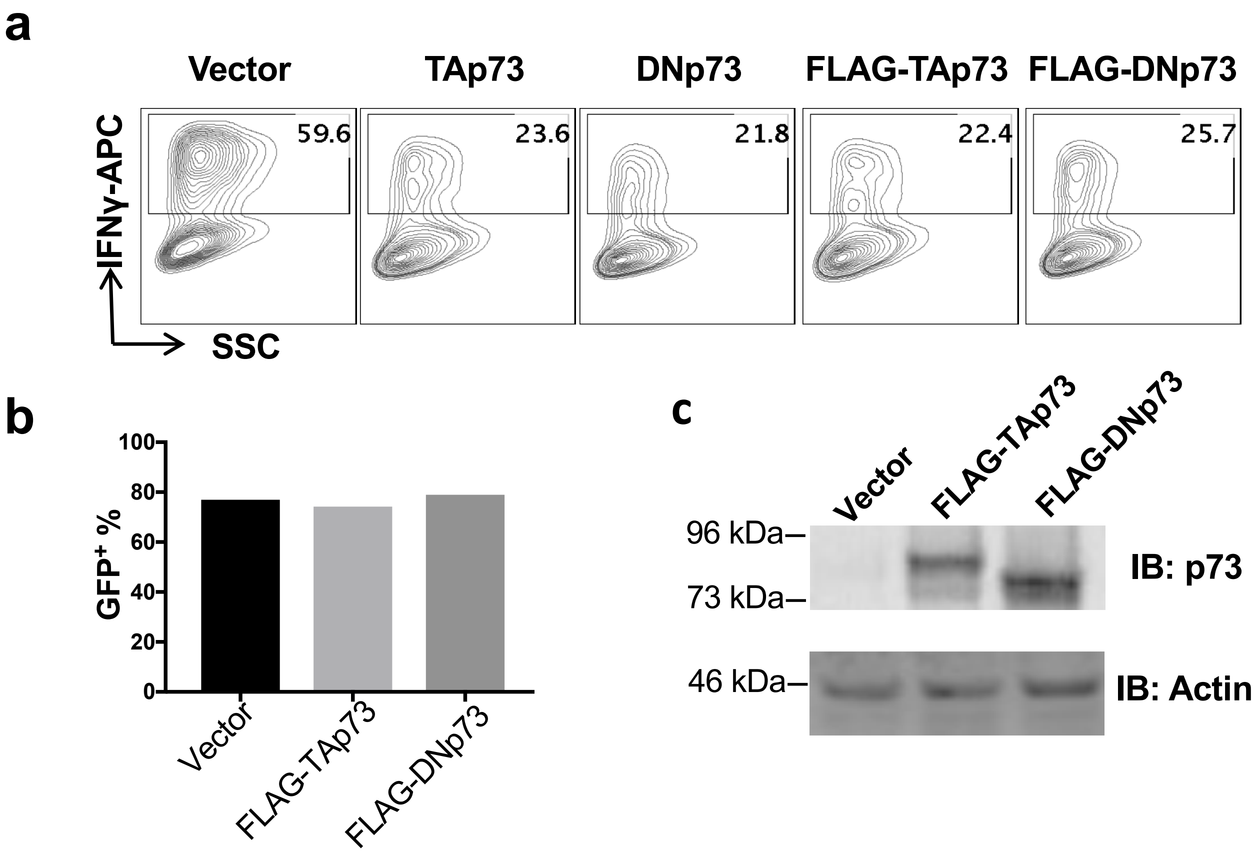


**Supplementary Fig. 3: N-terminal FLAG-tagged p73 proteins inhibits IFNγ expression in Th1 differentiated cells. a-c,** Empty vector, TAp73, DNp73, or FLAG-tagged versions of TAp73 and DNp73 were overexpressed in Th1 cells via retroviral transduction. Transduced cells were identified as $GFP^+$ cells, and IFNγ protein expression was assayed by flow cytometric staining and shown as a FACS plot (**a**). **b-c,** Empty vector, FLAG-TAp73 and FLAG-DNp73 were overexpressed in Th1 cells via retroviral transduction and the percentage of transduced cells ($GFP^+$) was determined by flow cytometric analysis **(b)** and FLAG-p73 expression level was measured by western blotting using p73 antibody **(c)**. A full scanned blot image is provided in **Supplemental Fig. 9c**. Data are from one representative of two independent experiments. Source data for **a-b** are provided in Source Data Files.

# Supplementary Figure 4

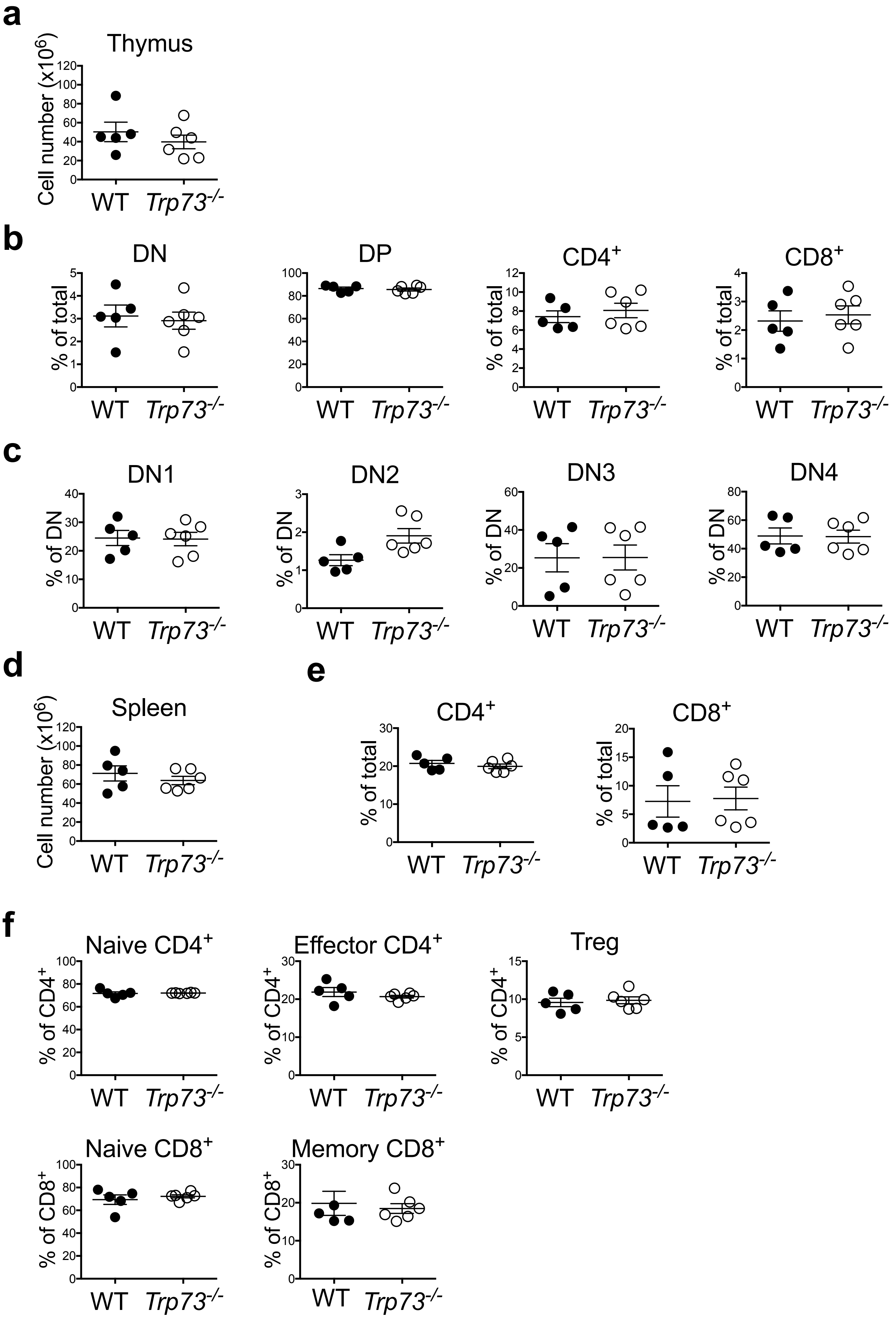

**Supplementary Fig. 4: Normal thymic and splenic T cells populations in *Trp73$^{-/-}$* mice. a-c,** Total cells were isolated from the thymus of *Trp73$^{-/-}$* mice and WT littermates and analyzed by flow cytometry. **a,** Total thymocyte numbers. **b**, Double negative (DN; $CD4^-CD8^-$), double positive (DP; $CD4^+CD8^+$), CD4 single positive (SP) and CD8 SP thymocytes (characterized by CD4 and CD8 expression). **c**, Percentage of DN1, DN2, DN3, and DN4 populations of DN ( $CD4^-CD8^-$) T cells characterized by CD44 and CD25 expression based on flow cytometry. **d-f,** Similarly**,** total cells were isolated from the spleen and analyzed by flow cytometry. Total splenocyte numbers are shown in (**d**). CD4 and CD8 T cell populations in the spleen are shown as percentages of total cells in (**e**). Different subpopulations of T cells were characterized based on CD44, CD62L, and CD25 expression; shown are naïve T cells ($CD44^{Lo}CD62L^{Hi}$), effector T cells ($CD44^{Hi}CD62L^{Lo}$) and Treg ($CD25^+$), and the percentage of each population is shown as indicated in (**f**). Data are pooled results from n = 2 independent experiments with n=5 (WT) and n=6 ( *Trp73$^{-/-}$*) mice per group in total. Data are presented as mean ± SEM in scatter dot plots. Source data for **a-f** are provided in a Source Data File.

# Supplementary Figure 5

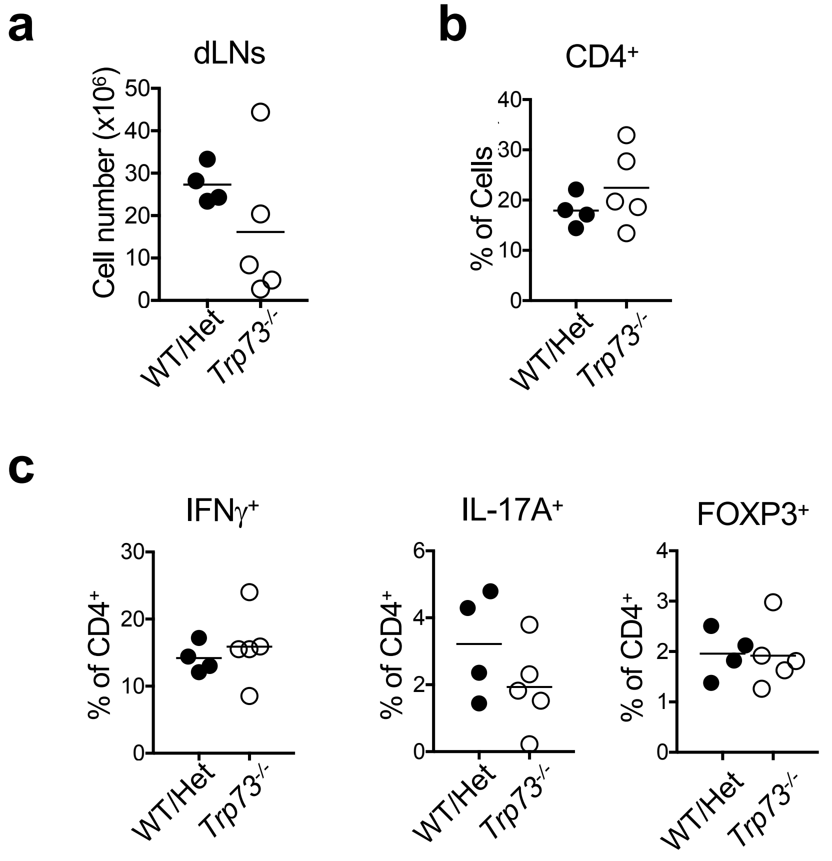


**Supplementary Fig. 5: Effect of *Trp73* gene deletion on different draining lymph node populations in EAE mice. a-c,** Total cells were isolated from draining lymph nodes (dLN) from WT/Het (n=4) or *Trp73*$^{-/-}$ (n=5) mice 27 days after EAE induction. Total recovered cell numbers (**a**) and the percentage of CD4$^+$ cells (**b**) are shown as indicated. **c,** Total cells from draining LNs were stimulated with $MOG_{35-55}$ for 48 h, and then IFNγ- or IL-17A-producing cells and FoxP3$^+$ cells were analyzed by flow cytometry. All data are presented as mean values in scatter dot plots. Data are from one representative of three experiments with 5 mice per group in each experiment. Source data for **a-c** are provided in a Source Data File.

# Supplementary Figure 6

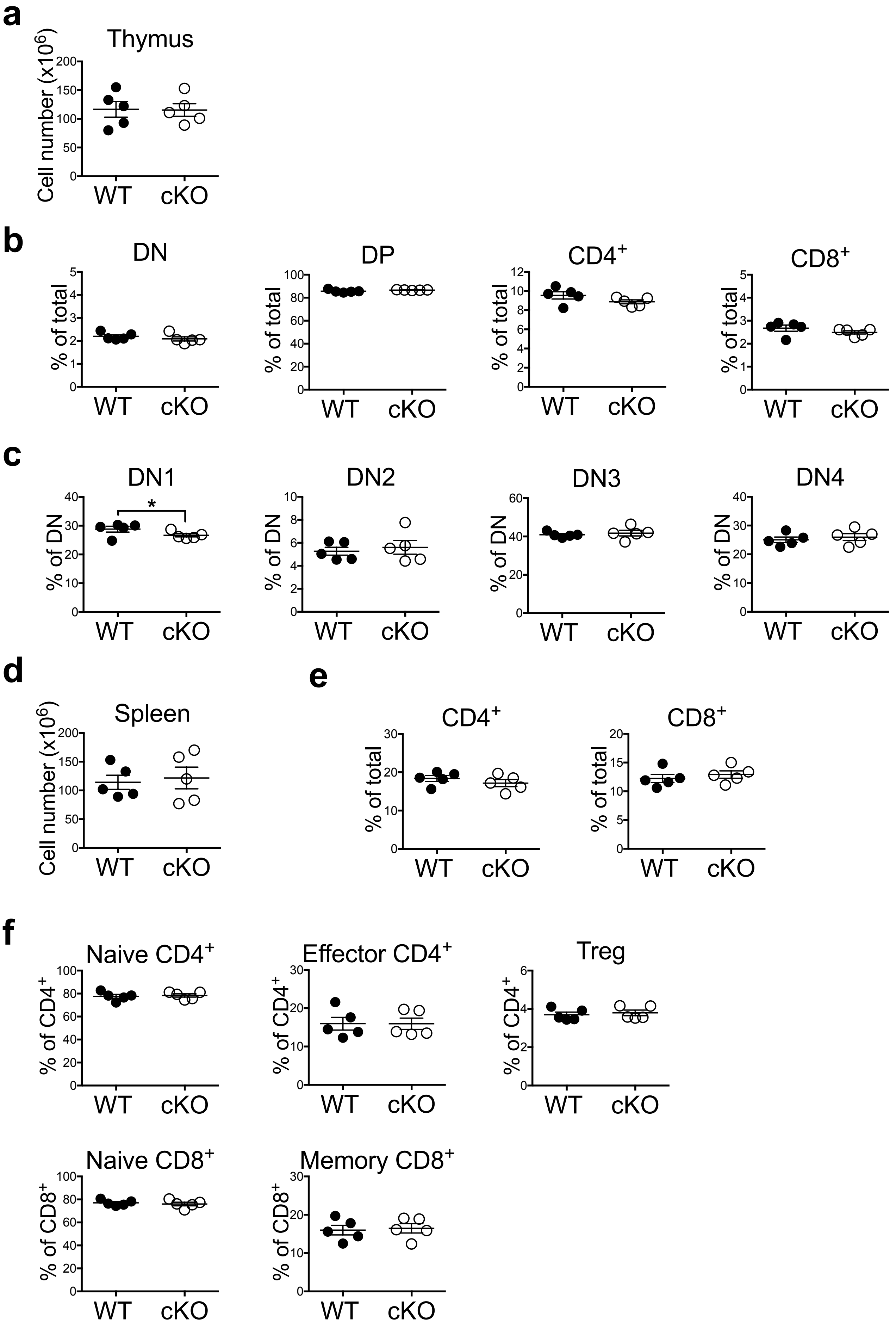

**Supplementary Fig. 6: Normal thymic and splenic T cells populations in mice with selective *Trp73$^{-/-}$* deletion in T cells. a-c,** Total cells were isolated from the thymus of *Trp73* cKO mice and WT littermates and analyzed by flow cytometry. **a,** Total thymocyte numbers. **b**, Double negative (DN; $CD4^{-}CD8^{-}$), double positive (DP; $CD4^{+}CD8^{+}$), CD4 single positive (SP) and CD8 SP thymocytes (characterized by CD4 and CD8 expression). **c**, Percentage of DN1, DN2, DN3, and DN4 populations of DN ( $CD4^{-}CD8^{-}$) T cells characterized by CD44 and CD25 expression based on flow cytometry. **d-f,** Similarly**,** total cells were isolated from the spleen and analyzed by flow cytometry. Total splenocyte numbers are shown in (**d**). CD4 and CD8 T cell populations in the spleen are shown as percentages of total cells in (**e**). Different subpopulations of T cells were characterized based on CD44, CD62L, and CD25 expression; shown are naïve T cells ($CD44^{Lo}CD62L^{Hi}$), effector T cells ($CD44^{Hi}CD62L^{Lo}$) and Treg ($CD25^{+}$), and the percentage of each population is shown as indicated in (**f**). Data are from one representative of n = 2 independent experiments, with n = 5 mice per group in each experiment. The results are shown as mean ± SEM in scatter dot plots. Source data for **a-f** are provided in a Source Data File.

# Supplementary Figure 7

**a**

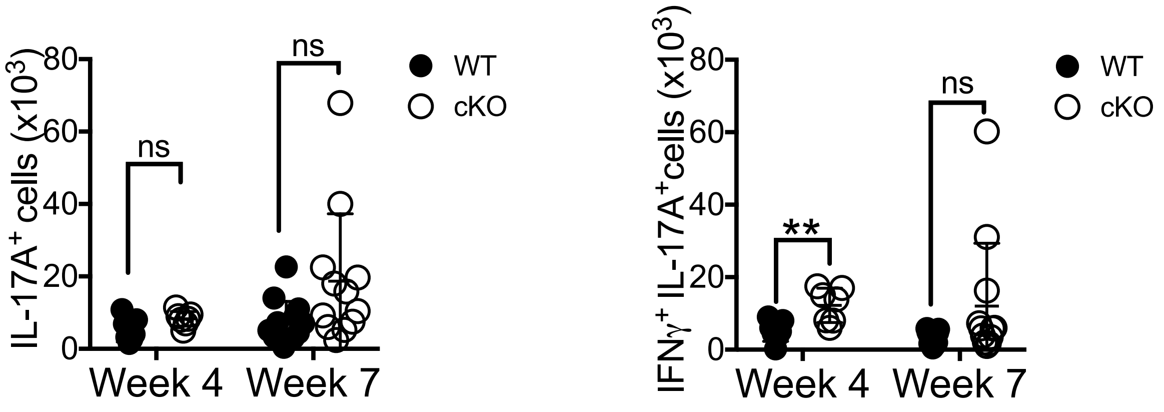


**b**

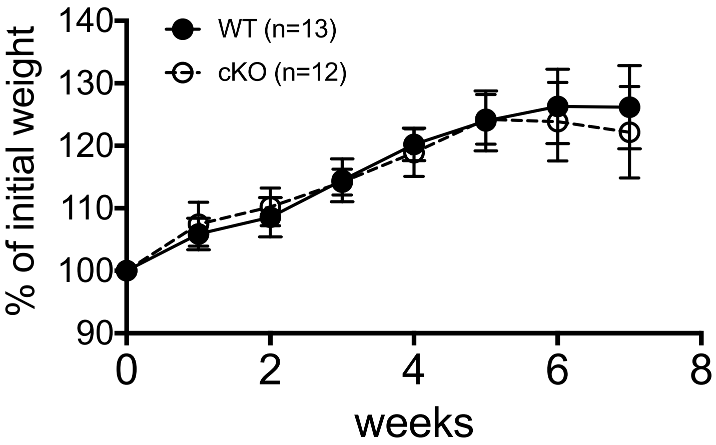


**Supplementary Fig. 7. IL-17A$^{+}$ and IFN-$\gamma^{+}$IL-17A$^{+}$ T cell responses and weight changes during adoptive-transfer colitis. a,** Total cells were isolated from the colons of individual mice, 4 and 7 weeks following adoptive transfer of naïve CD4$^{+}$ T cells to *Rag2*$^{-/-}$ mice, and stained for intracellular IL-17A and IFN-γ. Shown are total numbers of IL-17A single-positive and IFN-$\gamma^{+}$IL-17A$^{+}$ double positive T cells in mice transferred WT or cKO T cells. **b,** weight changes in *Rag2*$^{-/-}$ mice transferred WT or cKO T cells. Data are combined from two experiments with 9-10 mice per group in each experiment. In total, n=7 mice per group were analyzed at week 4, n=13 (WT) and n=12 (*Trp73* cKO) mice per group were analyzed at week 7. All data were presented as scatter dot plots with mean ± SD indicated and analyzed by two-tailed unpaired Student's t-test with Welch's correction, and the P values are indicated (ns, P>=0.05; *, P < 0.05; **, P < 0.01; ***, P < 0.001). Source data for **a-f** are provided in Source Data Files. Source data for **a** and **b** are provided in a Source Data File.

# Supplementary Figure 8

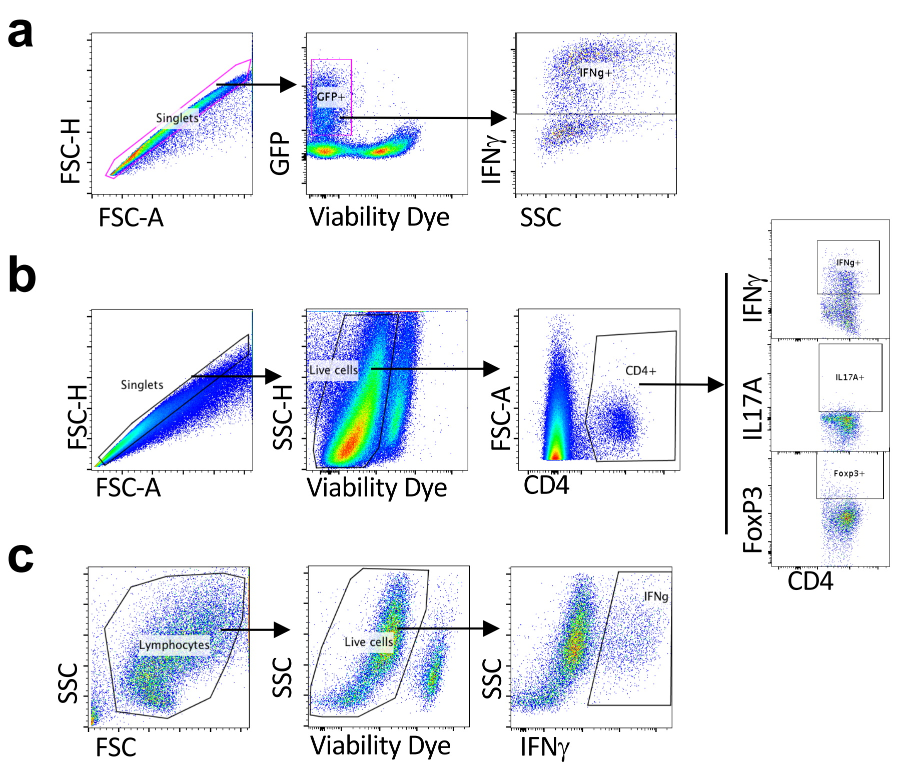


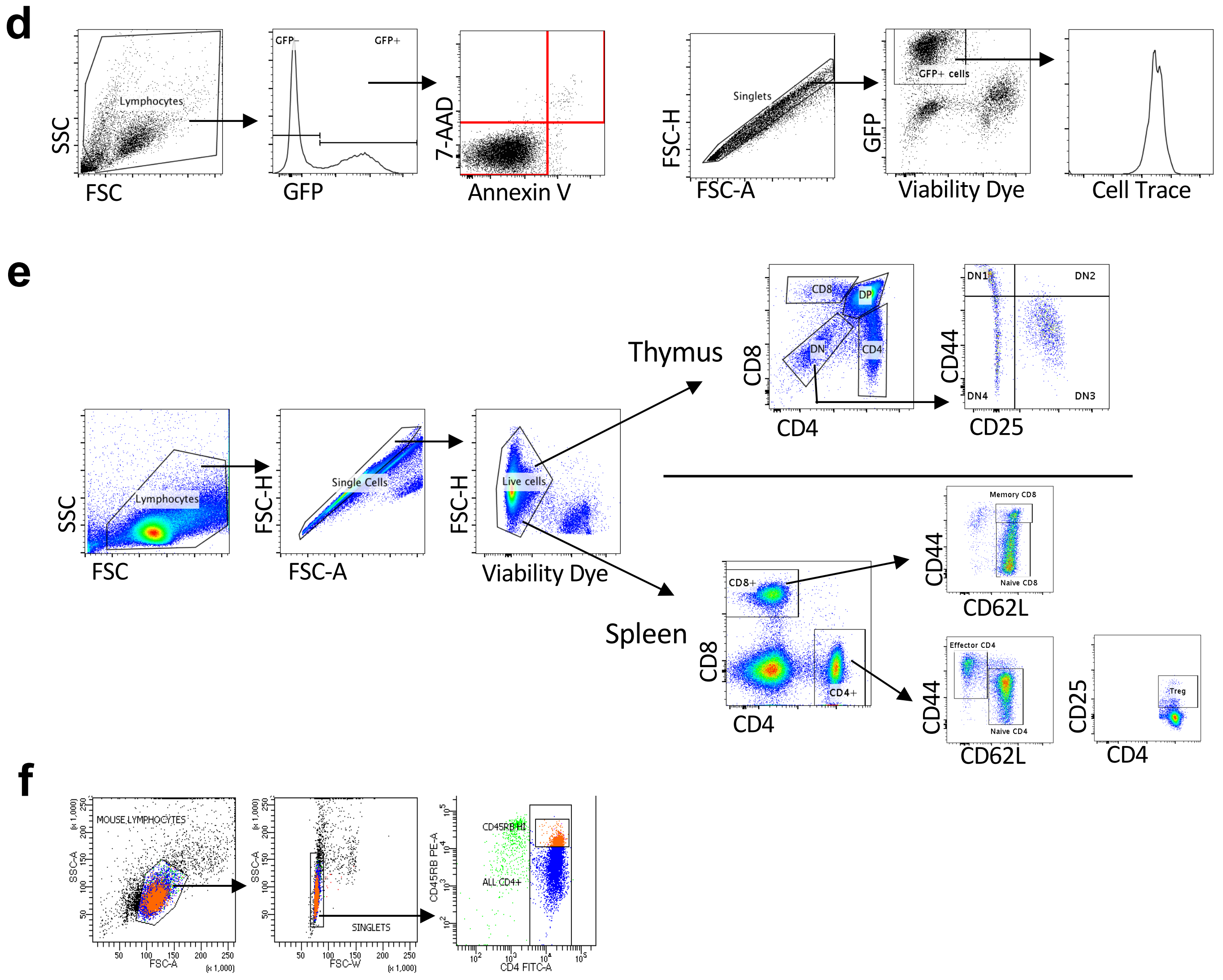


**Supplementary Fig. 8. Gating strategies used for flow cytometry analysis. a**, Gating strategy used to identify retrovirus transduced Th1 cells presented in **Fig. 1a-d**, **1f-k**, **Fig. 2b** and **Fig. 5e**. **b,** Gating strategy used to identify different infiltrated lymphocyte populations from spinal cord and dLNs presented in **Fig. 6c-d** and **Fig. S5**. **c,** Gating strategy used to analyze *in vitro* differentiated Th1 cells presented in **Fig. 7b**. **d,** Gating strategy used for analyzing cell death and proliferation rate in p73 overexpressed cells presented in **Fig. S2a-c**. **e,** Gating strategy used for analyzing general T cell populations in thymus and spleen presented in **Fig. S4** and **Fig. S6**. **f,** Gating strategy used for sorting out donor naïve ($CD4^{+}CD25^{-}CD45RB^{hi}$) T cells for transfer into *Rag2*$^{-/-}$ mice for IBD induction in **Fig. 7d-f** and **Fig. S7**.

## Supplementary Figure 9

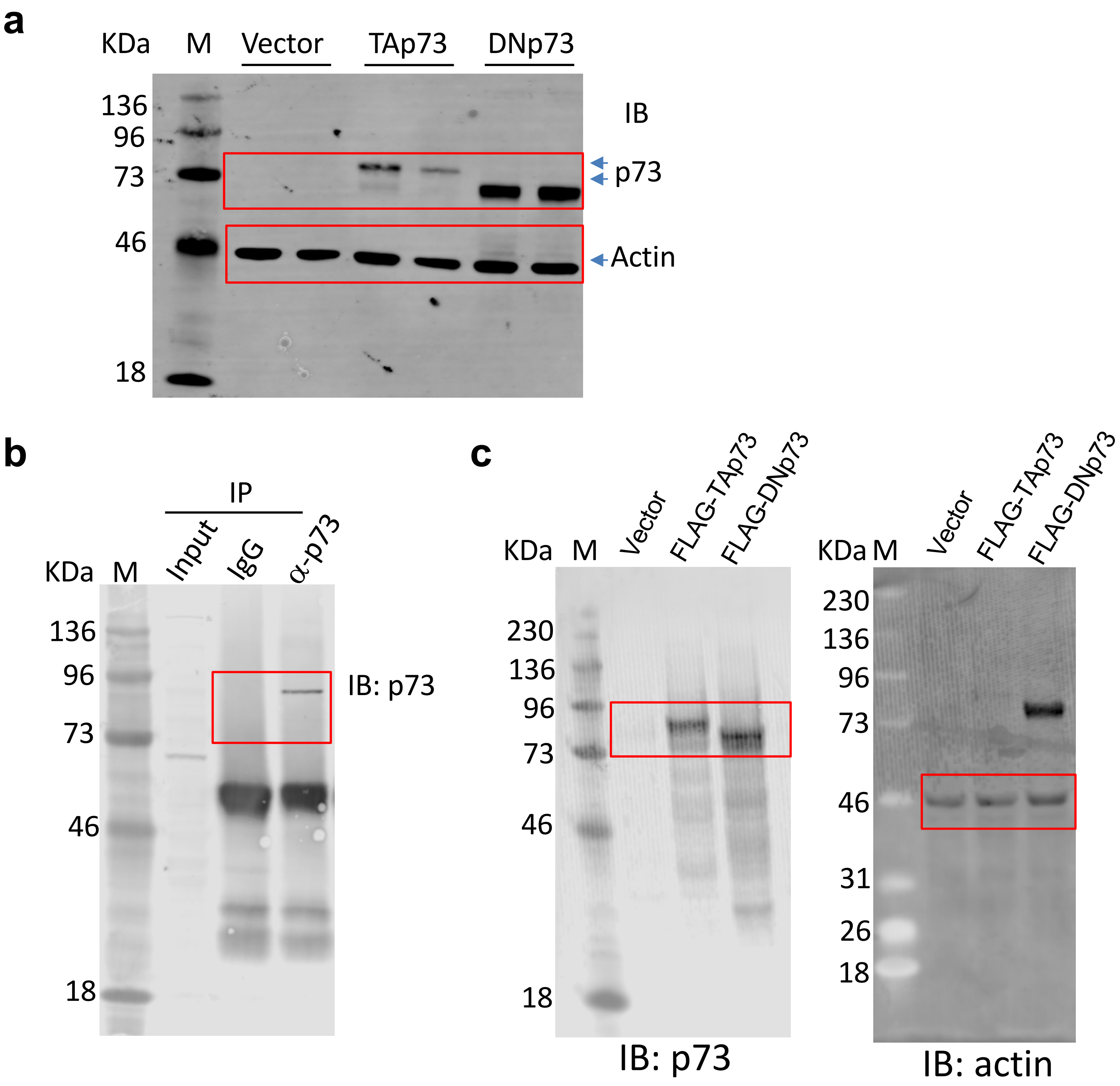


**Supplementary Fig. 9. Full size scan images of western blots. a-c,** Unprocessed western blots images for **Fig. 1e** (**a**), **Fig. 5a** (**b**) and **Fig. S3c** (**c**). Red boxes outlined the regions that were shown in the figures.

# Supplementary Table 1

| Gene | Score | Significant Time points | p-value | | | | | |
|---|---|---|---|---|---|---|---|---|
| | | | 0 hr | 4 hr | 16 hr | 24 hr | 48 hr | 72hr |
| *Rora* | 12.26 | 3 | | 1.85E-05 | 6.21E-04 | 4.76E-05 | | |
| *Stat1* | 11.00 | 3 | | 3.36E-05 | 5.45E-04 | | | 5.49E-04 |
| *Trp73* | 10.35 | 3 | 8.96E-04 | 7.22E-04 | | 6.98E-05 | | |
| | | | | | | | | |
| *Junb* | 10.49 | 2 | 2.66E-04 | 1.20E-07 | | | | |
| *Zbtb7c* | 8.75 | 2 | | 2.45E-04 | | 7.19E-06 | | |
| *Satb2* | 8.71 | 2 | | | 4.38E-05 | 4.49E-05 | | |
| *Vdr* | 8.69 | 2 | | | | 1.56E-05 | | 1.32E-04 |
| *Olig3* | 7.92 | 2 | | | 1.83E-05 | 6.63E-04 | | |
| *Npas2* | 7.82 | 2 | | | | 3.55E-05 | 4.23E-04 | |
| *Etv2* | 7.82 | 2 | | | | 3.02E-04 | 5.01E-05 | |
| *Glis3* | 7.79 | 2 | | 2.04E-04 | | 7.91E-05 | | |
| *Npas3* | 7.68 | 2 | | 3.03E-04 | | 6.98E-05 | | |
| *Foxm1* | 7.52 | 2 | | | 4.31E-05 | 7.02E-04 | | |
| *Runx2* | 7.40 | 2 | | | | 4.76E-05 | | 8.28E-04 |
| *Irf4* | 7.33 | 2 | | | 6.69E-04 | 6.98E-05 | | |
| *Tcf4* | 7.31 | 2 | | 2.87E-04 | | 1.72E-04 | | |
| *Foxd3* | 7.05 | 2 | | | 7.72E-04 | 1.14E-04 | | |
| *Mef2c* | 7.02 | 2 | 4.63E-04 | | | 2.05E-04 | | |
| *Stat4* | 7.02 | 2 | | 1.73E-04 | | | | 5.49E-04 |
| *Tcf12* | 6.98 | 2 | | 4.20E-04 | | 2.48E-04 | | |
| *Foxo3* | 6.92 | 2 | | | | 2.89E-04 | 4.16E-04 | |
| *Nr6a1* | 6.91 | 2 | | | | 2.67E-04 | | 4.66E-04 |
| *Pbx1* | 6.78 | 2 | 4.67E-04 | | | 3.51E-04 | | |
| *Uncx* | 6.72 | 2 | | | | 3.47E-04 | | 5.49E-04 |
| *Myt1l* | 6.69 | 2 | | | | 5.09E-04 | | 4.01E-04 |
| *Ebf1* | 6.56 | 2 | 7.29E-04 | | 3.81E-04 | | | |
| *Foxj2* | 6.54 | 2 | | | 5.70E-04 | 5.09E-04 | | |
| *Nr3c2* | 6.49 | 2 | | | | 3.61E-04 | | 9.03E-04 |
| *Hif1a* | 6.48 | 2 | | | | 5.41E-04 | | 6.11E-04 |
| *Esr1* | 6.44 | 2 | | | | 3.85E-04 | | 9.53E-04 |
| *Dlx2* | 6.40 | 2 | | | | 4.60E-04 | | 8.70E-04 |
| *Runx3* | 6.22 | 2 | | | | 6.84E-04 | | 8.79E-04 |

**Supplementary Table 1: Transcription factors identified by computational genetic analysis.** Shown are the transcription factors identified by HBCGM with significant P values based on correlating the allelic pattern with *Ifng* mRNA expression at the indicated time points ($P < 0.01$). *P* values were caculated using analysis of variance (ANOVA)-based statistical modeling.

# Supplementary Table 2

| Strain Name | Vendor | Stock Number |
|---|---|---|
| C57BL/6J | Jackson Laboratory | 664 |
| 129S1/SvImJ | Jackson Laboratory | 2448 |
| A/J | Jackson Laboratory | 646 |
| AKR/J | Jackson Laboratory | 648 |
| C3H/HeJ | Jackson Laboratory | 659 |
| DBA/2J | Jackson Laboratory | 671 |
| NOD/LtJ | Jackson Laboratory | 1976 |
| BALB/cJ | Jackson Laboratory | 651 |
| CBA/J | Jackson Laboratory | 654 |
| LP/J | Jackson Laboratory | 676 |
| SJL/J | Jackson Laboratory | 686 |
| MRL/MpJ | Jackson Laboratory | 486 |
| NZB/BlnJ | Jackson Laboratory | 684 |
| NZW/LacJ | Jackson Laboratory | 1058 |
| SM/J | Jackson Laboratory | 687 |
| FVB/NJ | Jackson Laboratory | 1800 |
| $Stat1^{\pm}$ | Jackson Laboratory | 12606 |
| $Stat4^{-/-}$ | Jackson Laboratory | 28526 |
| C57BL/6 $Rag2^{-/-}$ | Taconic Biosciences | RAGN12 |

**Supplementary Table 2: Commercial source mice strain stock number list.**

# Supplementary Table 3

| Gene Name | Direction | Sequence (5'-3') |
|---|---|---|
| *Mdm2* | Forward | GCCGCAGAATGACATCAG |
| | Reverse | GAGAGTCCCGATCATTCC C |
| | | |
| *Ifng* | Forward | AGTATGACAGCAATGATGCC |
| | Reverse | GGTCTGAGCAGGAAGTAGAG |
| | | |
| *Il12rb2*-A | Forward | TGTGTAGTGAAGACAGGCCC |
| | Reverse | ACGAGACTCGACAGCACAAC |
| | | |
| *Il12rb2*-B | Forward | TGTGTAGTGAAGACAGGCCC |
| | Reverse | ACGAGACTCGACAGCACAAC |
| | | |
| *Il2ra* | Forward | TGCAAAACATCTGTCCACTAC |
| | Reverse | AAGTCTTCTCTGACAGCCC |
| | | |
| *Tbx21* | Forward | TCCCCAGTGCCATCTCTCTC |
| | Reverse | CCTCCTTTTAGCGGAAAGCG |
| | | |
| Nonspecific site | Forward | AGTCCAGACAGCTTAGTGTCCACG |
| | Reverse | AGAACCAGGTGTAGGATTGCGGA |

**Supplementary Table 3: Primer sequences for ChIP-qPCR.** The sequence of primers (5’-3’) used in ChIP-qPCR assays are listed.
