# Supplementary material for: Transcription factor p73 regulates Th1 differentiation
Source: Nat Commun. 2020 Mar 19;11:1475. doi: 10.1038/s41467-020-15172-5 (PMC7081339; doi:10.1038/s41467-020-15172-5)
Supplement: Supplementary file 2 — Reporting Summary [file 41467_2020_15172_MOESM2_ESM.pdf]

nature research

Corresponding author(s): Warren J. Leonard

Last updated by author(s): Feb 12, 2020

Reporting Summary

Nature Research wishes to improve the reproducibility of the work that we publish. This form provides structure for consistency and transparency in reporting. For further information on Nature Research policies, see Authors & Referees and the Editorial Policy Checklist .

Statistics

For all statistical analyses, confirm that the following items are present in the figure legend, table legend, main text, or Methods section.

n/a	Confirmed	
[ ]	[x]	The exact sample size (*n*) for each experimental group/condition, given as a discrete number and unit of measurement
[ ]	[x]	A statement on whether measurements were taken from distinct samples or whether the same sample was measured repeatedly
[ ]	[x]	The statistical test(s) used AND whether they are one- or two-sided<br>*Only common tests should be described solely by name; describe more complex techniques in the Methods section.*
[x]	[ ]	A description of all covariates tested
[x]	[ ]	A description of any assumptions or corrections, such as tests of normality and adjustment for multiple comparisons
[ ]	[x]	A full description of the statistical parameters including central tendency (e.g. means) or other basic estimates (e.g. regression coefficient) AND variation (e.g. standard deviation) or associated estimates of uncertainty (e.g. confidence intervals)
[ ]	[x]	For null hypothesis testing, the test statistic (e.g. *F*, *t*, *r*) with confidence intervals, effect sizes, degrees of freedom and *P* value noted<br>*Give P values as exact values whenever suitable.*
[x]	[ ]	For Bayesian analysis, information on the choice of priors and Markov chain Monte Carlo settings
[x]	[ ]	For hierarchical and complex designs, identification of the appropriate level for tests and full reporting of outcomes
[x]	[ ]	Estimates of effect sizes (e.g. Cohen's *d*, Pearson's *r*), indicating how they were calculated

Our web collection on statistics for biologists contains articles on many of the points above.

Software and code

Policy information about availability of computer code

Data collection	1. Flow cytometry: BD FACS Diva v8.0.1<br>2. Western Blotting: Image Studio Lite, v5.2<br>3. qPCR, CFX Manager v3.1
Data analysis	1. Flow cytometry: FlowJo v10<br>2. Statistics and Data plotting: GraphPad Prism 7<br>3. NGS Data analysis and visualization: R v3.60 (with packages: edgeR v3.24.3, gglot2 v3.2.1, pheatmap v1.0.12), Bowtie v1.0.1, Tophat v2.2.1, IGVTools v2.2.10, RSEM v1.2, MACS v1.4, and Homer v4.10.<br>4. Statistical analysis on strain-specific effect: SAS v9.4.

For manuscripts utilizing custom algorithms or software that are central to the research but not yet described in published literature, software must be made available to editors/reviewers. We strongly encourage code deposition in a community repository (e.g. GitHub). See the Nature Research guidelines for submitting code & software for further information.

Data

Policy information about availability of data

All manuscripts must include a data availability statement. This statement should provide the following information, where applicable:

- Accession codes, unique identifiers, or web links for publicly available datasets
- A list of figures that have associated raw data
- A description of any restrictions on data availability

All relevant data are available from the authors. ChIP-Seq and RNA-Seq data sets have been deposited in the Gene Expression Omnibus under accession number GSE107368. T All other source data have been provided in a Source Data File ("Source Data Files.zip"). All the statistics source data for Figs. 1, 2b, 3d, 5a, 5b, 5d, 5e, 6, and 7 and Supplementary Figs. S1, S2b, S2d, S3b, S4, S5, S6, and S7 are provided as Prism (GraphPad software) files. The original flow cytometry data for Figs. 1a, 7b, 7f and Supplementary Figs. S2a, S2c, and S3a are provided in corresponding folders as Flow Cytometry Standard (FCS) files. Unprocessed western images for

Figs. 1e and 5a and Supplementary Fig. S3c are provided as PowerPoint (pptx) files.

Field-specific reporting

Please select the one below that is the best fit for your research. If you are not sure, read the appropriate sections before making your selection.

☒ Life sciences ☐ Behavioural & social sciences ☐ Ecological, evolutionary & environmental sciences

For a reference copy of the document with all sections, see nature.com/documents/nr-reporting-summary-flat.pdf

Life sciences study design

All studies must disclose on these points even when the disclosure is negative.

Sample size	No statistical method was used to predetermine sample size. For the EAE model, we included all available mice. For the IBD model, we followed standard protocols used by our collaborators, who are experts in this area. For all other experiments, we followed recommended sample size from well established protocols. All experiments were repeated, as indicated.
Data exclusions	No data was excluded from this study.
Replication	Biological replicates are included to ensure the reproducibility and all repeated experiments are successful. For in vitro experiments, the number of replicates are equal to individual mice used and are independently repeated as reported. For in vivo experiments, sample size per group and independently repeated experiments are described in the manuscript.
Randomization	For all in vitro experiments with retroviral transduction, same genotype mice are pooled then in vitro differentiated cells were equally allocated to different treatment groups. Mice are sex- and age- matched and are randomly assigned to different treatment and control groups. For the KO mice, control WT litter mates were used for comparisons.
Blinding	For all in vivo and individual mouse in vitro differentiation experiments, all the mice were coded and the data collection such as clinical score for EAE, or flow cytometery analysis was performed in blind fashion without the knowledge of the codes. For all in vitro experiments involving retroviral transductions, cells were pooled from the same genotype mice. Not all in vitro experiments were blinded but all were repeated as indicated. We do not believe that blinding is always necessary for these experiments nor is it always practical for all experiments in standard individual laboratories.

Reporting for specific materials, systems and methods

We require information from authors about some types of materials, experimental systems and methods used in many studies. Here, indicate whether each material, system or method listed is relevant to your study. If you are not sure if a list item applies to your research, read the appropriate section before selecting a response.

Materials & experimental systems

n/a	Involved in the study	
☐	☒	Antibodies
☐	☒	Eukaryotic cell lines
☒	☐	Palaeontology
☐	☒	Animals and other organisms
☒	☐	Human research participants
☒	☐	Clinical data

Methods

n/a	Involved in the study	
☐	☒	ChIP-seq
☐	☒	Flow cytometry
☒	☐	MRI-based neuroimaging

Antibodies

Antibodies used	PerCP/Cy5.5 anti-CD25 (Biolegend, Clone PC61), PE anti-IL12Rb2 (Miltenyi Biotec, Clone REA200), APC anti-IFNg (Biolegend, CloneXMG1.2), or BV421 anti-T-bet (Biolegend, Clone 4B10), anti-FLAG (M2) antibody (Sigma, clone M2), anti-p73 antibody (Abcam, cloneEP436Y), FITC anti-TCRb (eBioscience, Clone H57-597), PE/Cy5 and FITC anti-CD4(eBioscience, Clone GK1.5), APC anti-IL17A (eBioscience, Clone B7), PE anti-CD45RB (eBioscience, Clone C363.16A).
Validation	All fluorophore conjugated antibodies are against mouse protein and used for flow cytometry. Anti-FLAG antibody is not species specific and used for western blotting and immunoprecipitation. Anti-p73 antibody is against human and mouse protein, and was used for western blotting and immunoprecipitation. All antibodies used in this study are commercially available, and the validations were completed by the manufacturers.

Eukaryotic cell lines

Policy information about cell lines

Cell line source(s)	HEK293T was purchased from ATCC and p73 KO ES cells were purchased from the knockout consortium (www. Komp.org).
Authentication	None of the cell lines were authenticated.
Mycoplasma contamination	Cell lines were not tested for mycoplasma contamination.
Commonly misidentified lines (See ICLAC register)	N/A

Animals and other organisms

Policy information about studies involving animals; ARRIVE guidelines recommended for reporting animal research

Laboratory animals	Different inbred strains of mice (C57BL/6J, 129S1/SvImJ, A/J, AKR/J, C3H/HeJ, DBA/2J, NOD/LtJ, BALB/cJ, CBA/J, LP/J, SJL/J, MRL/MpJ, NZB/BlnJ, NZW/LacJ, SM/J, FVB/NJ), Stat1-/+ (Stat1tm1Dlv) and Stat4-/+ (Stat4em3Msasn) mice were purchased from the Jackson Laboratory. Trp73-/- (Trp73tm1a(KOMP)Wtsi) ES cells were purchased from the knockout consortium. The details of knockout strategy can be found at KOMP repository website (www. Komp.org) with the project ID: CSD89710. Trp73-/- mice were then generated from ES cells in the NHLBI transgenic core facility. Trp73f/f floxed mice (exon 5 was flanked with loxP sites) generated by Flp recombination from Trp73-/- mice. Then Trp73 conditional knockout mice (Trp73 cKO) were generated by crossing Trp73f/f mice with CD4-Cre mice. All Trp73-/- and Trp73 cKO were further backcrossed at least 4 generations to the C57BL/6 background.
Wild animals	No wild animals were used in this study.
Field-collected samples	No field-collected samples were used in the studies
Ethics oversight	Animal protocols were approved by the NHLBI Animal Care and Use Committee and followed the NIH Guidelines "Using Animals in Intramural Research."

Note that full information on the approval of the study protocol must also be provided in the manuscript.

ChIP-seq

Data deposition

- [x] Confirm that both raw and final processed data have been deposited in a public database such as GEO.
- [x] Confirm that you have deposited or provided access to graph files (e.g. BED files) for the called peaks.

Data access links *May remain private before publication.*	https://www.ncbi.nlm.nih.gov/geo/query/acc.cgi?acc=GSE107368
Files in database submission	GSM2865561 Vector<br>GSM2865562 TAp73 Over-expression<br>GSM2865563 DNp73 Over-expression<br>GSM2865564 Vector_FLAG<br>GSM2865565 DNp73_FLAG
Genome browser session (e.g. UCSC)	N/A

Methodology

Replicates	For p73 ChIP-seq, data shown are representative results from two biological replicates.
Sequencing depth	All reads from RNASeq and ChIPSeq were single end with sequencing lengths of 50bp.
Antibodies	Monoclonal anti-FLAG M2 antibody (Sigma, Clone M2).
Peak calling parameters	--pvalue=1e-5, --gsize=1.87e9, --shiftsize=100
Data quality	Mapping quality is provided in GEO data deposited
Software	Mapping (Bowtie v2.2.6), BAM and BED processing (bedtools v2.25.0), peak calling (MACS v1.4.2), differential gene expression (edgeR v3.24.3)

Flow Cytometry

Plots

Confirm that:

- [x] The axis labels state the marker and fluorochrome used (e.g. CD4-FITC).
- [x] The axis scales are clearly visible. Include numbers along axes only for bottom left plot of group (a 'group' is an analysis of identical markers).
- [x] All plots are contour plots with outliers or pseudocolor plots.
- [x] A numerical value for number of cells or percentage (with statistics) is provided.

Methodology

Sample preparation	Cells were stained with surface marker antibodies in FACS buffer (PBS+0.5%BSA), PerCP/Cy5.5 anti-CD25 (Biolegend, Clone PC61) or PE anti-IL12Rb2 (Miltenyi Biotec, Clone REA200). For intracellular staining, cells were fixed and permeabilized by Cytofix/ Cytoperm Buffer (BD Biosciences) after they were re-stimulated with 100 nM of PMA, 500 ng/ml of ionomycin, and BD GolgiPlug (BD Bioscience) for 4 h. Cells were then stained with isotype control antibodies, or APC anti-IFN (Biolegend, CloneXMG1.2), or BV421 anti-T-bet (Biolegend, Clone 4B10).
Instrument	BD LSRFortessa and BDFACSAriaTM cell sorter
Software	BD FACS Diva was used to collect data. FlowJo v10 was used to analyze the flow cytometry data.
Cell population abundance	The purity of isolated naive CD4+ T cells using Cd4+CD62+ T cells kit is >90%, as determined by flow cytometry.
Gating strategy	All gating strategies are included in the Source Data Files as FlowJo workspace (wsp) files and provided in the Supplementary information.

- [x] Tick this box to confirm that a figure exemplifying the gating strategy is provided in the Supplementary Information.
