# Supplementary material for: Transcription factor p73 regulates Th1 differentiation
Source: Nat Commun. 2020 Mar 19;11:1475. doi: 10.1038/s41467-020-15172-5 (PMC7081339; doi:10.1038/s41467-020-15172-5)
Supplement: Supplementary file 3 — Description of Additional Supplementary Files [file 41467_2020_15172_MOESM3_ESM.pdf]

Description of Additional Supplementary Files

Supplementary Data 1: Haplotype-based computational genetic analysis result. Shown are the significant P values of listed genes with haploblocks pattern that correlated with *Ifng* mRNA expression at the indicated time points ($P < 0.01$). *P* values were caculated using analysis of variance (ANOVA)-based statistical modeling.

Supplementary Data 2: RNA-Seq analysis results on Th1 cells transduced with p73 constructs or control vector. RPKM values of all mapped genes in alphabetical order based on RNA-Seq analysis of Th1 cells transduced with control vector, TAp73, or DNp73. Gene length (bp) is also shown. Negative Binomial distribution was used to calculate exact *P* values for differential expression and FDR was calculated using the Benjamini-Hochberg correction.

Supplementary Data 3: Differentially expressed genes in Th1 cells transduced with TAp73 versus vector. RPKM values of differentially expressed genes in Th1 cells transduced with TAp73 versus vector control. Log of the value of fold change (FC), P values, and false discovery rate (FDR) are shown. Negative Binomial distribution was used to calculate exact *P* values for differential expression and FDR was calculated using the Benjamini-Hochberg correction.

Supplementary Data 4: Differentially expressed genes in Th1 cells transduced with DNp73 versus vector. RPKM values of differentially expressed genes in Th1 cells transduced with DNp73 versus vector control. Log of fold change (FC), P values and false discovery rate (FDR) are shown. Negative Binomial distribution was used to calculate exact *P* values for differential expression and FDR was calculated using the Benjamini-Hochberg correction.

Supplementary Data 5: Genome wide p73 (TAp73 and DNp73) ChIP-Seq binding peaks in Th1 cells. Genomic locations of all p73 (TAp73 and DNp73) ChIP-Seq binding peaks and their assigned gene loci. Mapping was performed using HOMER software[44]. For each peak, *P* value was calculated using a dynamic Poisson distribution to capture local biases in read background levels and FDR values were calculated using the Benjamini-Hochberg correction.
